# FBXO2 Alleviates Intervertebral Disc Degeneration via Dual Mechanisms: Activating PINK1‐Parkin Mitophagy and Ubiquitinating LCN2 to Suppress Ferroptosis

**DOI:** 10.1002/advs.202506150

**Published:** 2025-08-12

**Authors:** Tongde Wu, Yanjin Wang, Beiduo Shen, Kai Guo, Ziqi Zhu, Yongzhou Liang, Jianhua Zeng, Desheng Wu

**Affiliations:** ^1^ Department of Spine Surgery Shanghai East Hospital School of Medicine Tongji University Shanghai 200092 China; ^2^ Department of Clinic of Spine Center Xinhua Hospital School of Medicine Shanghai Jiao Tong University 1665 Kongjiang Road Shanghai 200092 China; ^3^ Department of Nephrology Shanghai East Hospital School of Medicine Tongji University Shanghai 200092 China; ^4^ Department of Pediatric Cardiology West China Second University Hospital Sichuan University Chengdu Sichuan 610041 China; ^5^ Department of Pediatric Cardiology Xinhua Hospital School of Medicine Shanghai Jiao Tong University 1665 Kongjiang Road Shanghai 200092 China

**Keywords:** FBXO2, ferroptosis, intervertebral disc degeneration, LCN2, mitophagy

## Abstract

Intervertebral disc degeneration (IVDD), a leading cause of chronic low back pain, arises from nucleus pulposus (NP) cell dysfunction due to oxidative stress‐induced mitophagy impairment and ferroptosis, though regulatory mechanisms remain unclear. F‐box only protein 2 (FBXO2), a Kruppel‐like factor 10 (KLF10)‐regulated F‐box protein, is downregulated in degenerated human NP tissues and correlates with disease severity. Overexpression of FBXO2 restores extracellular matrix (ECM) homeostasis by promoting matrix component synthesis and inhibiting catabolic enzymes, while its knockdown exacerbates ECM degradation. Under oxidative stress, FBXO2 activates PTEN‐induced putative kinase 1 (PINK1)/Parkin‐dependent mitophagy, restoring mitochondrial membrane potential and reducing reactive oxygen species (ROS) accumulation. Proteomics reveals that FBXO2 suppresses ferroptosis by attenuating lipid peroxidation, glutathione depletion, and iron overload. Mechanistically, FBXO2 binds lipocalin‐2 (LCN2) via its FBA domain, promoting K27‐linked polyubiquitination to drive proteasomal degradation of this ferroptosis inducer. FBXO2 co‐expression reverses LCN2‐induced mitochondrial dysfunction and ferroptosis markers. In vivo, adeno‐associated virus 9 (AAV9)‐mediated overexpression of FBXO2 ameliorates IVDD in rats, whereas FBXO2 knockout (KO) mice exhibit exacerbated IVDD. LCN2 silencing in FBXO2‐deficient mice partially restores disc integrity and matrix component expression. These findings identify FBXO2 as a dual regulator coordinating mitophagy activation and ferroptosis suppression, offering therapeutic potential for IVDD progression.

## Graphical abstract

F‐box only protein 2 (FBXO2) combats intervertebral disc degeneration (IVDD) by coordinating dual protection. It activates PTEN‐induced putative kinase 1 (PINK1)‐Parkin mitophagy restoring mitochondrial function and targets lipocalin‐2 (LCN2) for degradation, suppressing ferroptosis. This rescues extracellular matrix homeostasis, reducing oxidative damage and disc degeneration in vivo. FBXO2 is a pivotal therapeutic target for IVDD progression.

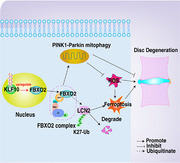

## 1 Introduction

The majority of individuals experience at least one episode of acute low back pain (LBP) in their lifetime, which frequently transitions into a chronic condition. Notably, over 60% of those suffering from mechanical back pain continue to experience pain or recurrences one year later.^[^ ^1^, ^2^ ^]^ A widely recognized contributor to low back pain is the degeneration of intervertebral discs, which underlies numerous degenerative disc conditions and poses significant socio‐economic burdens globally.^[^ ^3^, ^4^ ^]^ However, the precise pathogenesis and effective treatment for intervertebral disc degeneration (IVDD) are still under investigation. The intervertebral disc comprises three histologically distinct regions: the gelatinous nucleus pulposus (NP), the concentric lamellar annulus fibrosus (AF), and the cartilaginous endplates (EP). Among these components, NP cells play a pivotal role in synthesizing extracellular matrix constituents including collagen II and aggrecan, with their dysfunction and depletion constituting hallmark features of IVDD progression.^[^ ^5^ ^]^ The pathobiology of NP cell degeneration involves multifactorial interactions between aging processes and microenvironmental stressors, encompassing oxidative damage, biomechanical overload, and metabolic disturbances.^[^ ^6^, ^7^, ^8^ ^]^ Particularly, oxidative stress emerges as a central pathological driver through its disruption of mitochondrial homeostasis.^[^ ^9^, ^10^ ^]^

Reactive oxygen species (ROS), including superoxide (O_2_^−^), hydroxyl radicals (OH·), and hydrogen peroxide (H_2_O_2_), form critical mediators in IVDD pathogenesis.^[^ ^11^, ^12^ ^]^ Mitochondrial‐derived ROS engage in a self‐amplifying cycle with mitochondrial dysfunction, establishing a pathogenic feed‐forward loop.^[^ ^13^ ^]^ In this context, mitophagy serves as a crucial quality control mechanism that maintains mitochondrial homeostasis by selectively clearing damaged organelles, thereby effectively reducing pathological ROS accumulation.^[^ ^14^, ^15^ ^]^ The regulatory landscape of mitophagy encompasses several specific molecular mediators, with the PTEN‐induced putative kinase 1 (PINK1)‐Parkin axis representing the canonical pathway governing mitochondrial quality control.^[^ ^16^ ^]^ Notably, PINK1/Parkin‐mediated mitophagy has been mechanistically linked to the pathomechanisms underlying age‐associated degenerative disorders, particularly IVDD.^[^ ^17^, ^18^ ^]^ Nevertheless, the precise regulatory dynamics of mitophagy in NP cells under oxidative stress conditions remain incompletely understood.

Emerging evidence implicates ferroptosis – an iron‐dependent cell death pathway driven by Fenton reaction‐mediated lipid peroxidation – in IVDD pathobiology. This regulated necrosis modality manifests four cardinal features: 1) redox‐active iron accumulation, 2) glutathione peroxidase 4 (GPX4) inactivation, 3) antioxidant system collapse, and 4) membrane‐destructive lipid peroxide accumulation.^[^ ^19^, ^20^ ^]^ Mechanistic studies reveal that iron overload exacerbates disc degeneration through dual induction of oxidative stress and ferroptosis in endplate chondrocytes,^[^ ^21^ ^]^ while mechanical stress potentiates iron influx via Piezo1 channels to promote NP cell ferroptosis.^[^ ^22^ ^]^ Importantly, mitophagy deficiency creates a permissive microenvironment for ferroptosis through ROS‐mediated mitochondrial damage, establishing an intersectional mechanism driving NP cell dysfunction and IVDD progression.^[^ ^11^, ^23^, ^24^ ^]^ These findings position mitophagy regulation and ferroptosis inhibition as promising therapeutic targets.

**Scheme 1:**
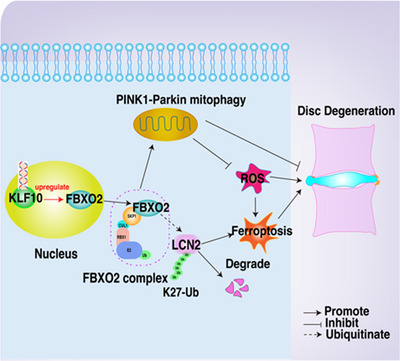
FBXO2 attenuates IVDD via dual‐target activation of mitophagy and suppression of ferroptosis.

Maintenance of cellular proteostasis critically depends on the ubiquitin‐proteasome system (UPS), a primary pathway for the selective degradation of proteins.^[^ ^25^ ^]^ This process is initiated by ubiquitination, a post‐translational modification involving the covalent conjugation of ubiquitin molecules to target substrates. The specificity of ubiquitin‐dependent degradation is largely conferred by E3 ubiquitin ligases, which recognize specific substrates.^[^ ^26^ ^]^ Within the extensive family of E3 ligases, cullin–RING ligases (CRLs) represent the predominant class. Among CRLs, the SKP1–Cullin 1–F‐box protein (SCF) complexes are the most extensively studied.^[^ ^27^ ^]^ The substrate recognition specificity of SCF complexes is determined by their interchangeable F‐box protein components. F‐box only protein 2 (FBXO2), an F‐box protein containing a conserved carbohydrate‐binding domain,^[^ ^28^ ^]^ serves as a critical substrate recognition component of the SCF E3 ubiquitin ligase complex.^[^ ^29^ ^]^ This multifunctional enzyme regulates diverse cellular processes including cell cycle progression, differentiation, and apoptosis through phosphorylation‐dependent substrate ubiquitination.^[^ ^30^, ^31^ ^]^ Despite its broad biological relevance, the functional significance of FBXO2 in IVDD pathogenesis remains largely unexplored. Our study establishes that FBXO2, transcriptionally activated by Kruppel‐like factor 10 (KLF10), restores PINK1/Parkin‐mediated mitophagy to sustain mitochondrial homeostasis and counteract oxidative stress in human NP cells. Mechanistically, FBXO2 orchestrates K27‐linked polyubiquitination and proteasomal degradation of lipocalin 2 (LCN2), thereby mitigating LCN2‐triggered ferroptosis and mitochondrial impairments during IVDD. Complementary in vivo studies demonstrate that FBXO2 overexpression attenuates disc degeneration in rodent IVDD models, whereas FBXO2 deletion exacerbates disease progression through LCN2‐mediated ferroptotic pathways. These findings collectively delineate FBXO2 as a critical regulator of intervertebral disc homeostasis through coordinated mitophagy enhancement and ferroptosis suppression (**Scheme** **1**).

## 2 Results

### 2.1 FBXO2, Positively Regulated by KLF10, Exhibited Decreased Expression in Degenerated NP Tissues and Correlated Significantly with IVDD Pathogenesis

Previous studies have established KLF10's pivotal role in IVDD pathogenesis.^[^ ^32^ ^]^ To elucidate its mechanistic involvement, we performed RNA sequencing and subsequently identified eight candidate transcriptional targets through chromatin immunoprecipitation (ChIP) assays (Figure S1A–C, Supporting Information). Among these, FBXO2 showed the most significant enrichment, with its mRNA levels markedly elevated following KLF10 overexpression (Figure S1D,E, Supporting Information).

Bioinformatic analysis using the JASPER database revealed four potential KLF10 binding sites within the FBXO2 promoter region: site 1 (‐916/‐895), site 2 (‐669/‐649), site 3 (‐534/‐369), and site 4 (‐182/‐122) (Figure S1F,G: Supporting Information). Dual‐luciferase reporter assays demonstrated that mutations in sites 1 and 2 caused significantly greater reductions in FBXO2 promoter activity compared to other sites, indicating their essential role in KLF10‐mediated transcriptional activation (Figure S1H, Supporting Information). The immunofluorescence (IF) analysis revealed distinct subcellular localization patterns: KLF10 predominantly localized to nuclei, while FBXO2 exhibited dual cytoplasmic and nuclear expression (Figure S1J, Supporting Information). Functional validation showed that KLF10 overexpression upregulated FBXO2 expression, whereas KLF10 knockdown produced the opposite effect (Figure S1I‐L, Supporting Information). These findings collectively underscore the critical role of KLF10 in regulating FBXO2 expression and provide valuable insights into the mechanisms underlying IVDD pathogenesis.

**Figure 1:**
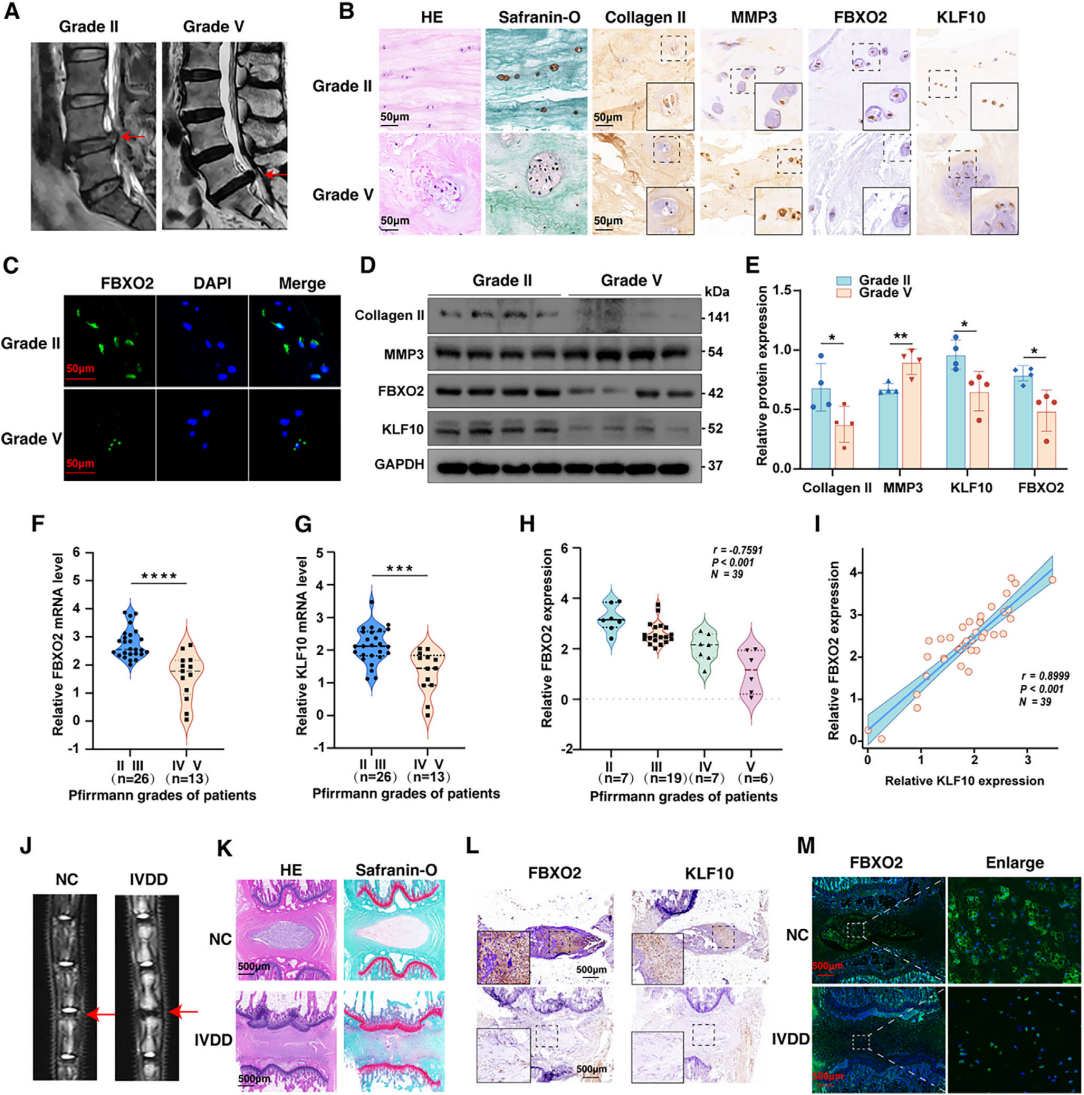
F‐box only protein 2 (FBXO2) was downregulated in degenerated nucleus pulposus (NP) tissues and significantly correlated with intervertebral disc degeneration (IVDD) pathogenesis. A) Human NP tissues were classified into Pfirrmann grades II and V based on T2‐weighted magnetic resonance imaging (MRI). B) Representative hematoxylin and eosin (H&E), Safranin‐O and Fast Green (SO&FG), and immunohistochemistry (IHC) staining (collagen II, matrix metallopeptidase 3 (MMP3), FBXO2, and Kruppel‐like factor 10 (KLF10)) of human NP tissues from Grade II and V discs. *n* = 3. Scale bars: 50 µm. C) The immunofluorescence (IF) staining of FBXO2 on human NP tissues from Grade II and V discs. n = 3. Scale bars: 50 µm. D,E) The protein levels of collagen II, MMP3, FBXO2, and KLF10 detected by Western blot (WB) in human NP tissues from Grade II and Grade V discs. *n* = 4. F,G) The mRNA levels of FBXO2 and KLF10 detected by quantitative reverse transcription polymerase chain reaction (qRT‐PCR) in human NP tissues from discs of different grades (Grade II and III, *n* = 26; Grade IV and V, *n* = 13). H,I) The correlation of FBXO2 with the Pfirrmann grades, determined by the Spearman's rank correlation coefficient (*r* = −0.7591, *n* = 39, *P* < 0.001), and with KLF10, determined by the Pearson's correlation coefficient (*r* = 0.8999, *n* = 39, *P* < 0.001). J) MR images of control and IVDD groups of rats. *n* = 3. K‐M) Representative H&E, SO&FG, IHC (FBXO2 and KLF10), and immunofluorescence (IF) staining (FBXO2) of rat discs. *n* = 3. Scale bars: 500 µm. All data are shown as the mean ± SD. Two‐tailed unpaired Student's *t*‐tests (E,F, G), Spearman's rank‐order analysis (H), and Pearson correlation analysis (I) were used to determine the statistical significance. * for *P* < 0.05, ** for *P* < 0.01, *** for *P* < 0.001, **** for *P* < 0.0001, NS for no significance.

To investigate the clinical relevance of FBXO2 in IVDD, we analyzed its expression in degenerated NP tissues from human patients and a rat model. Human NP specimens (n = 39; demographic details in Table S1, Supporting Information) were classified by Pfirrmann grades (**Figure** **1**A). The staining with hematoxylin and eosin (H&E) and Safranin‐O and Fast Green (SO&FG) revealed significant disorganization and calcification of the NP matrix, accompanied by chondroid‐like proliferation and clustering of NP cells, especially in tissues from the severely degenerated group (Grade V) (Figure 1B).^[^ ^33^ ^]^ Notably, the severe degeneration group exhibited low expression of the extracellular matrix protein collagen II, coupled with high expression of matrix metallopeptidase 3 (MMP3), which are hallmark features of intervertebral disc degeneration (Figure 1B,D,E). Immunohistochemistry (IHC), IF staining, and Western blot (WB) analyses demonstrated that, compared to the mild degeneration group (Grade II), FBXO2 expression was decreased in the severe degeneration group (Grade V) (Figure 1B‐E). Furthermore, the expression of FBXO2 was negatively correlated with the Pfirrmann grades of the IVDD patients, while positively correlated with the expression of KLF10 (Figure 1H,I). In a rat IVDD model induced by needle puncture (validated by magnetic resonance imaging (MRI) and histological staining), FBXO2 expression was similarly reduced in degenerative rat NP tissues (Figure 1J‐M). The concordant expression patterns of FBXO2 and KLF10 in both human and rat degenerated tissues further support their functional association (Figure 1B–G,J–L).

**Figure 2:**
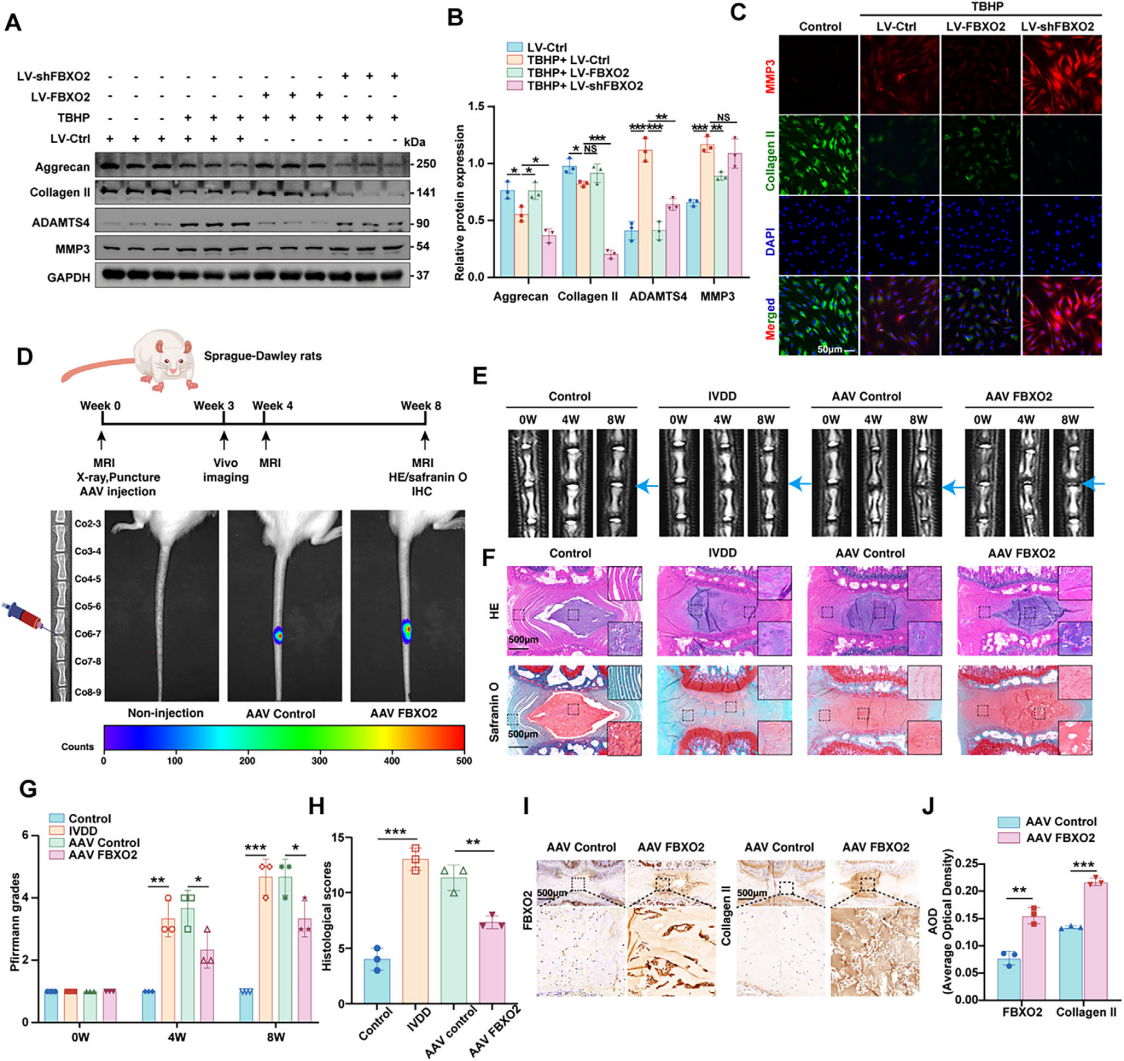
FBXO2 regulated extracellular matrix (ECM) homeostasis to suppress intervertebral disc degeneration (IVDD) progression in vitro and in vivo. A,B) The protein levels of aggrecan, collagen II, a disintegrin and metalloproteinase with thrombospondin 4 (ADAMTS4), and matrix metallopeptidase 3 (MMP3) determined by Western blot (WB). C) The protein levels of collagen II and MMP3 determined by immunofluorescence (IF). Scale bars: 50 µm. D) A flowchart of the experiments in rats. The Representative In Vivo Imaging System (IVIS) Spectrum images exhibited the biodistribution of D‐luciferin in normal rats and rats with IVDD that have been treated with either AAV9 expressing a control or overexpressing FBXO2. E,G) T2‐weighted MRI images of the indicated groups obtained at 0 weeks, 4 weeks, and 8 weeks after surgery, and Pfirrmann grades calculated. F,H) Hematoxylin and eosin (H&E) and Safranin‐O and Fast Green (SO&FG) staining performed on the intervertebral discs of the indicated groups at 8 weeks after surgery, and subsequently, histological scores analyzed. Scale bars: 500 µm. I,J) The immunohistochemistry (IHC) staining for FBXO2 and collagen II performed on the disc samples of each group, and the average optical density (AOD) quantified. Scale bars: 500 µm. All data are shown as the mean ± SD. Two‐tailed unpaired Student's t‐tests J) and one‐way analysis of variance (ANOVA) were used followed by Tukey's post hoc test (B,G, H) to determine the statistical significance. * for *P* < 0.05, ** for *P* < 0.01, *** for *P* < 0.001, NS for no significance. *n* = 3.

Given the crucial role of oxidative stress in the pathophysiological mechanisms of IVDD, we employed tert‐butyl hydroperoxide (TBHP), an exogenous ROS generator, to establish an in vitro model of disc degeneration. As depicted in Figure S2A,B, Supporting Information, minimal alterations in FBXO2 expression were observed following treatment with low concentrations of TBHP (less than 50 × 10^−6^ m). Conversely, a pronounced decrease in FBXO2 expression was evident at a higher concentration of 75 × 10^−6^ m. Furthermore, time‐course experiments revealed a gradual decline in FBXO2 expression over time. Therefore, we selected 75 × 10^−6^ m TBHP for subsequent experiments, as this concentration effectively suppressed FBXO2 expression and cell viability after a 2‐h treatment period, while aligning with oxidative stress levels documented in human IVDD models and minimizing cytotoxic effects (**Figures** **2**A–C and S5C, Supporting Information). Both interleukin‐1β (IL‐1β) and TBHP are well‐established inducers of IVDD in cellular models, as extensively documented in the literature.^[^ ^34^, ^35^ ^]^ IL‐1β mimics the inflammatory microenvironment characteristic of IVDD, while TBHP models oxidative stress—a key driver of NP cell senescence and ferroptosis.^[^ ^36^ ^]^ Significantly, under stimulation with IL‐1β, FBXO2 expression decreased in a time‐ and concentration‐dependent manner, similar to the effect observed with TBHP (Figure S2C,D, Supporting Information). This concordance indicates that FBXO2 is a common responsive factor in IVDD pathogenesis, irrespective of whether the trigger is inflammatory or oxidative.

### 2.2 FBXO2 Regulated (Extracellular Matrix) ECM Homeostasis to Suppress IVDD Progression in Vitro and in Vivo

To explore the potential correlation between FBXO2 expression and the functional characteristics of human NP cells, we performed lentiviral transduction using three distinct vectors: a control lentivirus (LV‐Ctrl), an FBXO2‐specific shRNA‐expressing lentivirus (LV‐shFBXO2), and an FBXO2‐overexpressing lentivirus (LV‐FBXO2). The transduction efficiency of these viruses was evaluated through WB analysis (Figure S2E,F, Supporting Information). Based on the WB results, shFBXO2‐26 was chosen for further investigation, aiming to knockdown FBXO2 expression. Functional assays revealed that FBXO2 overexpression significantly downregulated catabolic markers MMP3 and a disintegrin and metalloproteinase with thrombospondin 4 (ADAMTS4) while upregulating anabolic ECM components collagen II and aggrecan. Conversely, FBXO2 knockdown produced opposing effects, establishing a bidirectional regulatory relationship between FBXO2 expression and NP cell homeostasis (**Figure** **2**A–C).

To investigate the therapeutic potential of FBXO2 in a rat model of IVDD, we conducted an experiment involving the administration of adeno‐associated virus 9 (AAV9) into the rat intervertebral disc to overexpress FBXO2 following IVDD surgery. Initially, X‐ray imaging was used to identify the (coccygeal 6–7) Co6‐7 level for the establishment of the IVDD model. Subsequently, we performed intradiscal injections of either AAV9‐expressing FBXO2 or control AAV9 immediately following IVDD induction. The efficacy of FBXO2 expression via AAV9 in the rat discs was confirmed through in vivo small animal imaging (Figure 2D). To address potential off‐target effects of localized AAV9 delivery, we conducted comprehensive histological analyses of major organ systems. H&E staining revealed no significant morphological alterations in the brain, heart, lung, liver, or kidney tissues of either AAV9‐control or AAV9‐FBXO2 treated animals (Figure S3D, Supporting Information). Critically, immunohistochemical analysis confirmed the absence of ectopic FBXO2 expression in these peripheral tissues (Figure S3D, Supporting Information), ruling out systemic transgene dissemination following intradiscal injection. We then evaluated therapeutic efficacy through longitudinal MRI examinations at baseline (time 0), and at 4 and 8 weeks post‐surgery across all groups. As shown in Figure 2E–G, at both 4 and 8 weeks post‐surgery, the Pfirrmann grades of the rat intervertebral discs were significantly higher in the IVDD group compared to the control group. However, overexpression of FBXO2 significantly lowered the degenerative degree score. All rats were euthanized at the 8‐week mark, and subsequent pathological staining, including H&E, SO&FG, and IHC, was performed. The histological scores of the rat discs in the IVDD group were higher than those in the control group. In contrast, overexpression of FBXO2 resulted in lower histological scores compared to the AAV9 control group (Figure 2F,H). Furthermore, IHC results indicated successful overexpression of FBXO2 in the AAV9‐FBXO2 group, accompanied by upregulation of the extracellular matrix protein collagen II (Figure 2I,J). These integrated in vitro and in vivo data establish FBXO2 as a critical regulator of IVDD pathogenesis through modulation of ECM homeostasis and catabolic processes.

**Figure 3:**
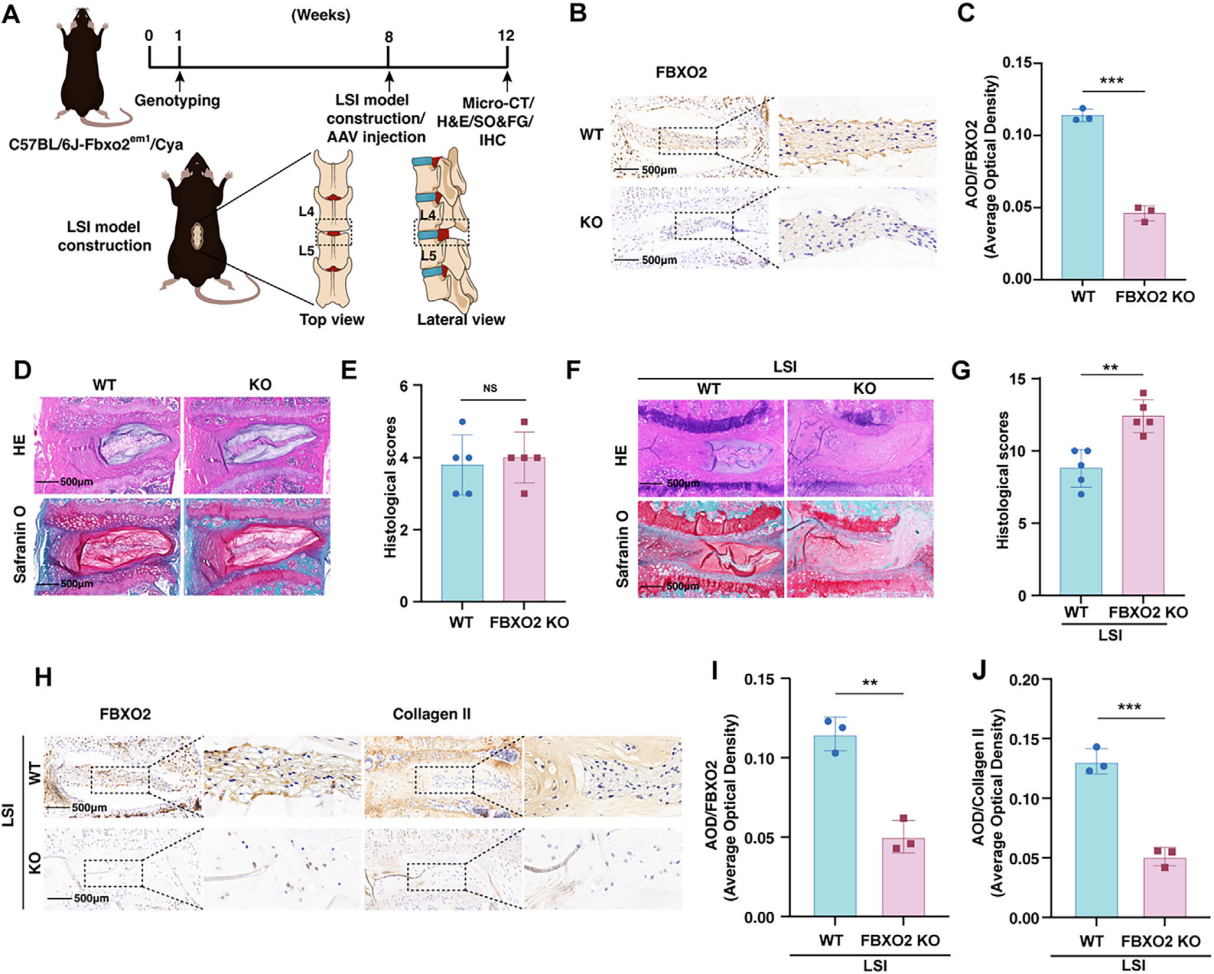
FBXO2 knockout (KO) exacerbated intervertebral disc degeneration (IVDD) progression. A) A flowchart depicting the experiments conducted in mice, including lumbar spine instability (LSI) model construction. B,C) The immunohistochemistry (IHC) staining for FBXO2 performed on mice of both wild‐type (WT) and KO genotypes. *n* = 3. D–G) Hematoxylin and eosin (H&E) and Safranin‐O and Fast Green (SO&FG) staining performed on WT and KO mice with or without LSI model construction, and the histological scores analyzed. *n* = 5. Scale bars: 500 µm. H–J) The IHC staining for FBXO2 and collagen II performed on WT and KO mice with LSI model construction. *n* = 3. Scale bars: 500 µm. All data are shown as the mean ± SD. Two‐tailed unpaired Student's *t*‐tests (C,E,G,I, J) to determine the statistical significance. * for *P* < 0.05, ** for *P* < 0.01, *** for *P* < 0.001, NS for no significance.

To further validate FBXO2 function in vivo, we established a lumbar IVDD mouse model via lumbar spine instability (LSI) methodology and generated FBXO2 knockout (KO) mice using CRISPR‐Cas9 technology (**Figure** **3**A). FBXO2 was successfully ablated, as confirmed by IHC analysis (Figure 3B,C). To comprehensively evaluate systemic impacts of global FBXO2 ablation, we first examined 3‐month‐old naive mice without IVDD induction. FBXO2 KO mice displayed comparable gross morphology and body weight to wild‐type (WT) controls (Figure S3A,B, Supporting Information). We next performed histological assessment of major organ systems in LSI‐operated mice. H&E staining revealed no pathological alterations in brain, heart, lung, liver, or kidney tissues of KO versus WT littermates (Figure S3C, Supporting Information). Critically, IHC confirmed efficient ablation of FBXO2 in all examined peripheral organs of KO animals (Figure S3C, Supporting Information), validating tissue‐specific knockout without compensatory expression. In mice without IVDD induction, FBXO2 KO showed no significant alterations in lumbar discs compared to controls (Figure 3D,E). Notably, FBXO2 KO mice exhibited accelerated IVDD progression, demonstrating significantly elevated histological degeneration scores versus WT littermates (Figure 3F,G). Molecular analysis revealed that FBXO2 deficiency downregulated collagen II expression levels compared with the WT group (Figure 3H,J).

### 2.3 FBXO2‐PINK1/Parkin Axis Restored Mitophagic Flux to Counteract Mitochondrial Dysfunction in IVDD

**Figure 4:**
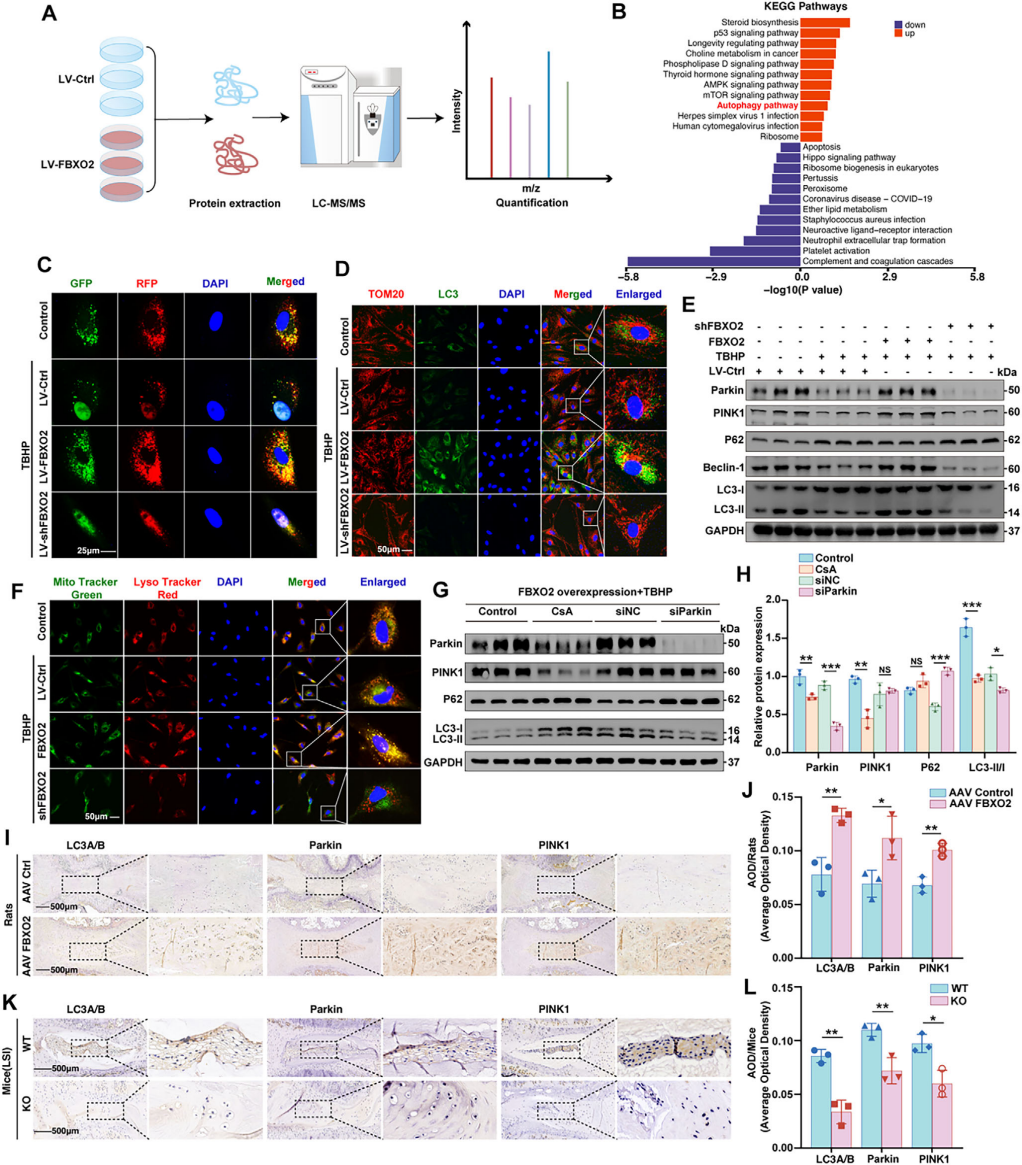
FBXO2‐PTEN‐induced putative kinase 1(PINK1)/Parkin axis restored mitophagic flux to counteract mitochondrial dysfunction in intervertebral disc degeneration (IVDD). A) Quantitative proteomic analysis of the lentivirus‐control (LV‐Ctrl) and LV‐FBXO2‐transfected human nucleus pulposus (NP) cells. B) The kyoto encyclopedia of genes and genomes (KEGG) pathway enrichment analysis identified significantly enriched pathways. C) AdV‐mRFP‐GFP‐LC3 used to monitor autophagy flux. Autophagosomes are labeled with both GFP and mRFP, resulting in a yellow appearance, whereas autolysosomes are labeled solely with mRFP and appear red in the merged image. Scale bars: 25 µm. D) The immunofluorescence (IF) showed co‐localization of LC3 and TOM20. Scale bars: 50 µm. E) The protein levels of PINK1, Parkin, P62, Beclin‐1 and LC3‐II/LC3‐I ratio determined by Western blot (WB). F) The colocalization of mitochondria with lysosomes visualized using the MitoTracker Green and LysoTracker Red assays. Scale bars: 50 µm. G,H) The protein levels of PINK1, Parkin, P62, and LC3‐II/LC3‐I ratio determined by WB. I,J) The immunohistochemistry (IHC) staining for LC3A/B, Parkin, and PINK1 performed on NP from rat models treated with AAV9 vectors expressing either a control construct or an FBXO2‐overexpressing construct, and the average optical density (AOD) quantified. Scale bars: 500 µm. K,L) The IHC staining for LC3A/B, Parkin and PINK1 performed on wild‐type (WT) and knockout (KO) mice with lumbar spine instability (LSI) model construction. Scale bars: 500 µm. All data are shown as the mean ± SD. Two‐tailed unpaired Student's *t*‐tests (J,L) and one‐way analysis of variance (ANOVA) were used followed by Tukey's post hoc test (H) to determine the statistical significance. * for *P* < 0.05, ** for *P* < 0.01, *** for *P* < 0.001, NS for no significance. *n* = 3.

To elucidate the molecular mechanisms underlying FBXO2‐mediated regulation of NP cell function, we performed quantitative proteomic profiling of LV‐Ctrl and LV‐FBXO2‐transfected human NP cells (**Figure** **4**A). Kyoto encyclopedia of genes and genomes (KEGG) pathway enrichment analysis identified significant upregulation of proteins associated with autophagy, p53 signaling, and longevity regulation pathways, coupled with downregulation of apoptosis, peroxisome function, and ether lipid metabolism pathways following FBXO2 overexpression (Figure 4B). To monitor autophagy flux, we infected the human NP cells with the mRFP‐GFP‐LC3 adenovirus (AdV‐mRFP‐GFP‐LC3). In the acidic lysosomal environment, autolysosomes appeared as red puncta due to the quenching of GFP, whereas autophagosomes emitted yellow signals resulting from the combined presence of both mRFP and GFP.^[^ ^37^ ^]^ We performed complementary IF co‐localization staining for the autophagosome formation marker LC3 and the mitochondrial outer membrane marker TOM20. As illustrated in Figure 4C,D, human NP cells with FBXO2 overexpression exhibited a greater number of red and yellow puncta and increased co‐localized fluorescence signals of TOM20 and LC3 under oxidative stress conditions, whereas inhibition of FBXO2 had the opposite effect. These data indicate that FBXO2 modulates autophagy in human NP cells under oxidative stress, although the precise molecular mechanism requires further investigation.

Given that oxidative stress induces mitochondrial dysfunction in human NP cells, we conducted a comprehensive analysis of multiple mitochondrial parameters. Specifically, we investigated the mitochondrial membrane potential (5,5′,6,6′‐Tetrachloro‐1,1′,3,3′‐tetraethylbenzimidazolylcarbocyanine iodide (JC‐1) and Tetramethylrhodamine ethyl ester (TMRE) assay), intracellular ROS levels, and mitochondrial‐specific ROS (MitoSOX) levels. Additionally, we utilized both MitoTracker Red and MitoTracker Green assays, which stain mitochondria in live cells. Notably, the accumulation of MitoTracker Red is contingent upon membrane potential, whereas MitoTracker Green accumulation is independent of it. Consequently, the ratio of MitoTracker Red to Green staining provides an indication of the proportion of healthy mitochondria. Our results showed that TBHP treatment significantly decreased mitochondrial membrane potential and the ratio of MitoTracker Red to Green staining, while simultaneously increasing ROS and MitoSOX levels in human NP cells (Figure S4A–F and S5D, Supporting Information). For visualization, we employed the MitoTracker Red probe to assess mitochondrial morphology. The MitoTracker Red assay demonstrated that normal cells exhibited an elongated mitochondrial network, whereas TBHP‐treated cells displayed mitochondrial fragmentation and accumulation around the nucleus (Figure S4G, Supporting Information). These findings indicated that TBHP induced mitochondrial dysfunction in human NP cells. Of particular interest, overexpression of FBXO2 had a protective effect on mitochondria. Specifically, it significantly increased mitochondrial membrane potential and the ratio of MitoTracker Red to Green staining, improved mitochondrial morphology, and suppressed the elevation of ROS and MitoSOX levels in human NP cells. However, the knockdown of FBXO2 reversed these beneficial alterations (Figure S4A–J, Supporting Information). These results suggested that FBXO2 mitigated mitochondrial damage and enhanced mitochondrial activity that was impaired by TBHP‐induced oxidative stress.

Although FBXO2 has demonstrated the ability to maintain mitochondrial homeostasis in human NP cells, whether it regulates oxidative stress‐induced mitophagy impairment—which plays a crucial role in IVDD—remains to be elucidated. WB revealed that FBXO2 overexpression activated mitophagy via the PINK1/Parkin signaling axis, with opposing effects observed upon FBXO2 knockdown (Figure 4E and Figure S4K, Supporting Information). Mitochondrial‐lysosomal colocalization assays using MitoTracker Green and LysoTracker Red confirmed increased mitochondrial clearance in FBXO2‐overexpressing cells under TBHP treatment, with reduced colocalization areas in FBXO2‐knockdown cells (Figure 4F). To elucidate the role of PINK1 and Parkin in mitophagy induced by the overexpression of FBXO2, human NP cells were pretreated with Cyclosporine A (CsA), a known inhibitor of mitophagy, or transfected with Parkin‐specific siRNA (Figure S2G, Supporting Information). The administration of CsA effectively inhibited the upregulation of PINK1 and Parkin protein levels, as well as the LC3‐II/LC3‐I ratio (Figure 4G,H). In contrast, when compared with cells treated with control siRNA, those transfected with Parkin‐siRNA exhibited a significant reduction in the LC3‐II/LC3‐I ratio, accompanied by an elevation in P62 expression. These findings were further corroborated by double‐labeled IF staining of TOM20 and Parkin (Figure S4L, Supporting Information). Notably, mitophagy can proceed through PRKN‐dependent pathways or receptor‐mediated alternative pathways (e.g., BNIP3/NIX). In this study, oxidative stress substantially downregulated BNIP3/NIX protein levels in human NP cells; however, neither FBXO2 overexpression nor knockdown significantly altered their expression levels under oxidative stress conditions (Figure S4M,N, Supporting Information). To investigate the role of FBXO2 in mitophagy regulation in animal models, IHC staining was performed on intervertebral disc tissues from rat and mouse IVDD models. AAV9‐FBXO2‐treated discs exhibited markedly elevated LC3A/B, Parkin, and PINK1 protein levels in the NP compared to controls, whereas FBXO2‐KO mouse discs showed pronounced attenuation of these mitophagy markers (Figure 4I‐L).

Collectively, these data demonstrate that FBXO2 preserves mitochondrial homeostasis in NP cells by selectively activating PINK1/Parkin‐mediated mitophagy, thereby mitigating oxidative stress‐induced mitochondrial dysfunction and attenuating IVDD progression both in vitro and in vivo.

### 2.4 FBXO2 Regulated the Oxidative Stress‐Induced Ferroptosis

**Figure 5:**
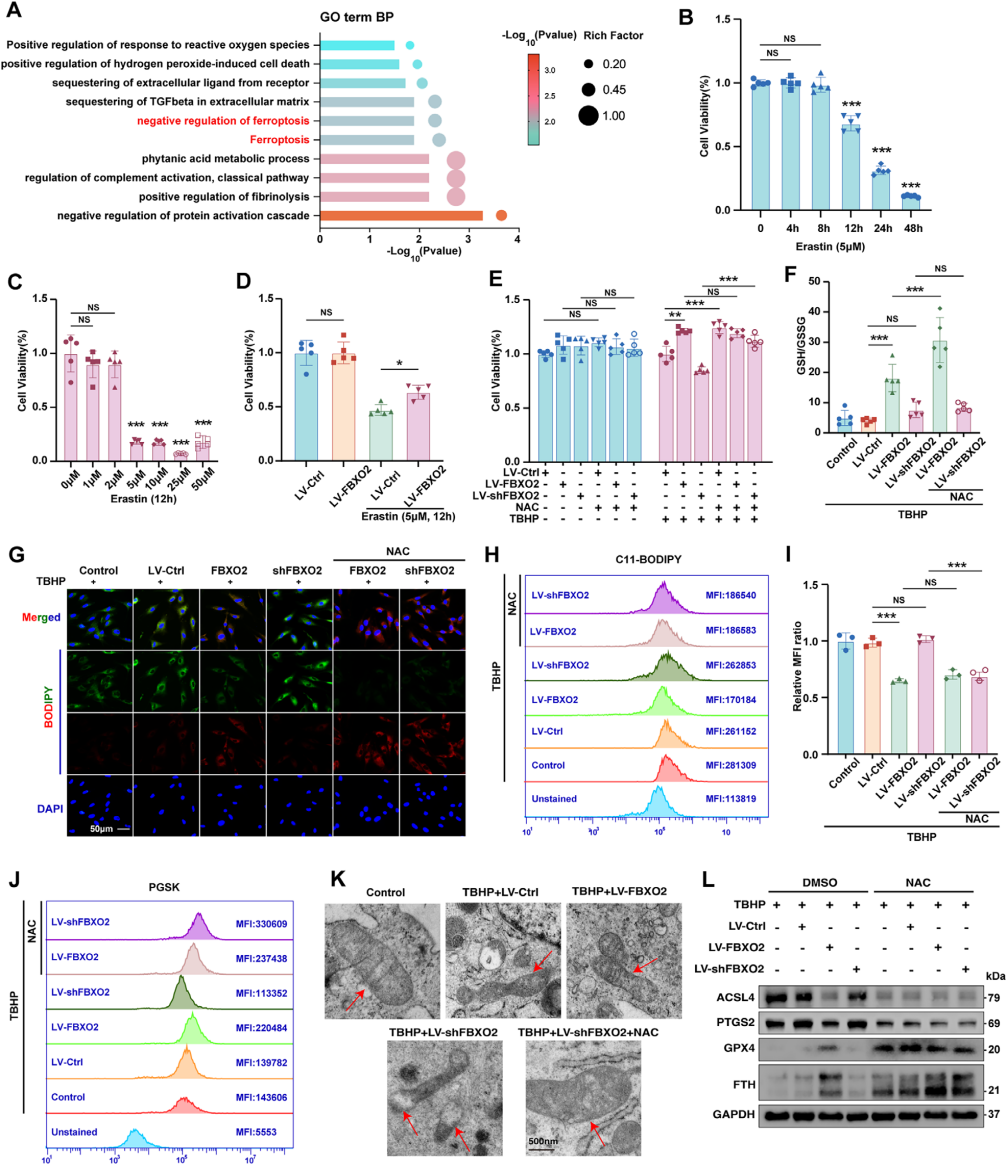
FBXO2 suppressed oxidative stress‐induced ferroptosis in human nucleus pulposus (NP) cells. A) Proteomic analysis showed significantly enriched gene ontology (GO) terms following FBXO2 overexpression. B–E) Cell proliferation detected by using the cell viability assay. *n* = 5. F) The glutathione (GSH) content detected by its assay, and the GSH/ glutathione disulfide (GSSG) ratio determined. *n* = 5. G–I) Lipid reactive oxygen species (ROS) production assessed using the C11‐BODIPY probe staining method, as well as by flow cytometry. *n* = 3. Scale bars: 50 µm. J) Intracellular ferrous iron levels measured using the Phen Green SK (PGSK) assay, and the results assessed by flow cytometry. *n* = 3. K) Representative transmission electron microscope (TEM) images of human NP cells in indicated groups. Red arrows indicate mitochondria. *n* = 3. Scale bars: 500 nm. L) The protein levels of ACSL4, PTGS2, glutathione peroxidase 4 (GPX4), and FTH detected by Western blot (WB) in human NP cells. N‐acetylcysteine (NAC) was utilized to inhibit ROS through its antioxidant properties. All data are shown as the mean ± SD. Two‐tailed unpaired Student's t‐tests (B,C) and one‐way analysis of variance (ANOVA) were used followed by Tukey's post hoc test (D,E,F, I) to determine the statistical significance. * for *P* < 0.05, ** for *P* < 0.01, *** for *P* < 0.001, NS for no significance.

Subsequent proteomic analysis demonstrated that significantly enriched gene ontology (GO) terms were mechanistically linked to ferroptosis regulatory pathways following FBXO2 overexpression (**Figure** **5**A). Emerging evidence indicates that oxidative stress may trigger and potentiate ferroptosis mechanisms in NP cells, consequently contributing to the progression of IVDD.^[^ ^22^, ^38^ ^]^ IHC analysis demonstrated significantly lower expression levels of GPX4 and FTH in the severe degeneration group (Grade V) compared to the mild degeneration group (Grade II), suggesting a potential association between ferroptosis and IVDD (Figure S5A,B: Supporting Information). To elucidate the regulatory role of FBXO2 in this process, we systematically investigated its effects on oxidative stress‐driven ferroptosis in NP cells. We first assessed the cytotoxic effects of two ferroptosis inducers, erastin and TBHP, on human NP cells. Both agents exhibited time‐ and concentration‐dependent suppression of cell viability, with TBHP showing comparable potency to the canonical ferroptosis inducer erastin (Figure 5B,C and Figure S5C, Supporting Information). Notably, FBXO2 overexpression effectively counteracted the viability loss caused by erastin or TBHP treatment (Figure 5D,E). To confirm the oxidative stress dependency of this ferroptotic process, we employed N‐acetylcysteine (NAC), a potent thiol‐based antioxidant. NAC treatment markedly restored cell viability in TBHP‐challenged cells, supporting the critical role of redox imbalance in ferroptosis execution. Subsequent mechanistic studies revealed that TBHP treatment disrupted mitochondrial membrane potential, as evidenced by reduced TMRE fluorescence intensity, while simultaneously elevating lipid peroxidation levels detected via BODIPY C11 oxidation (Figure S5D,E, Supporting Information). Parallel assays demonstrated that TBHP treatment induced glutathione (GSH) depletion and intracellular iron accumulation (measured by Phen Green SK (PGSK) assay), both of which are hallmark features of ferroptosis (Figure S5F,G: Supporting Information). Strikingly, FBXO2 overexpression attenuated all TBHP‐induced pathological changes, whereas FBXO2 knockdown exacerbated these effects. Importantly, NAC administration independently rescued mitochondrial membrane potential, restored GSH levels, and suppressed both lipid peroxidation and iron overload, regardless of FBXO2 expression status (Figure 5F–J and Figure S5I, Supporting Information). Transmission electron microscope (TEM) provided ultrastructural evidence of ferroptosis, showing characteristic mitochondrial abnormalities in TBHP‐treated cells, including fragmentation, vacuolization, shrinkage, and cristae disassembly (Figure 5K). These morphological alterations were aggravated by FBXO2 knockdown but ameliorated by either FBXO2 overexpression or NAC co‐treatment. WB analysis further confirmed that TBHP activated ferroptotic pathways, as evidenced by the downregulation of GPX4 and FTH, and the upregulation of ACSL4 and PTGS2. Consistent with functional rescue experiments, this effect was reversed by either FBXO2 overexpression or NAC intervention (Figure 5L and Figure S5H, Supporting Information).

### 2.5 FBXO2 Interacted with LCN2 and Triggered K27‐Linked Ubiquitination and Degradation of LCN2

**Figure 6:**
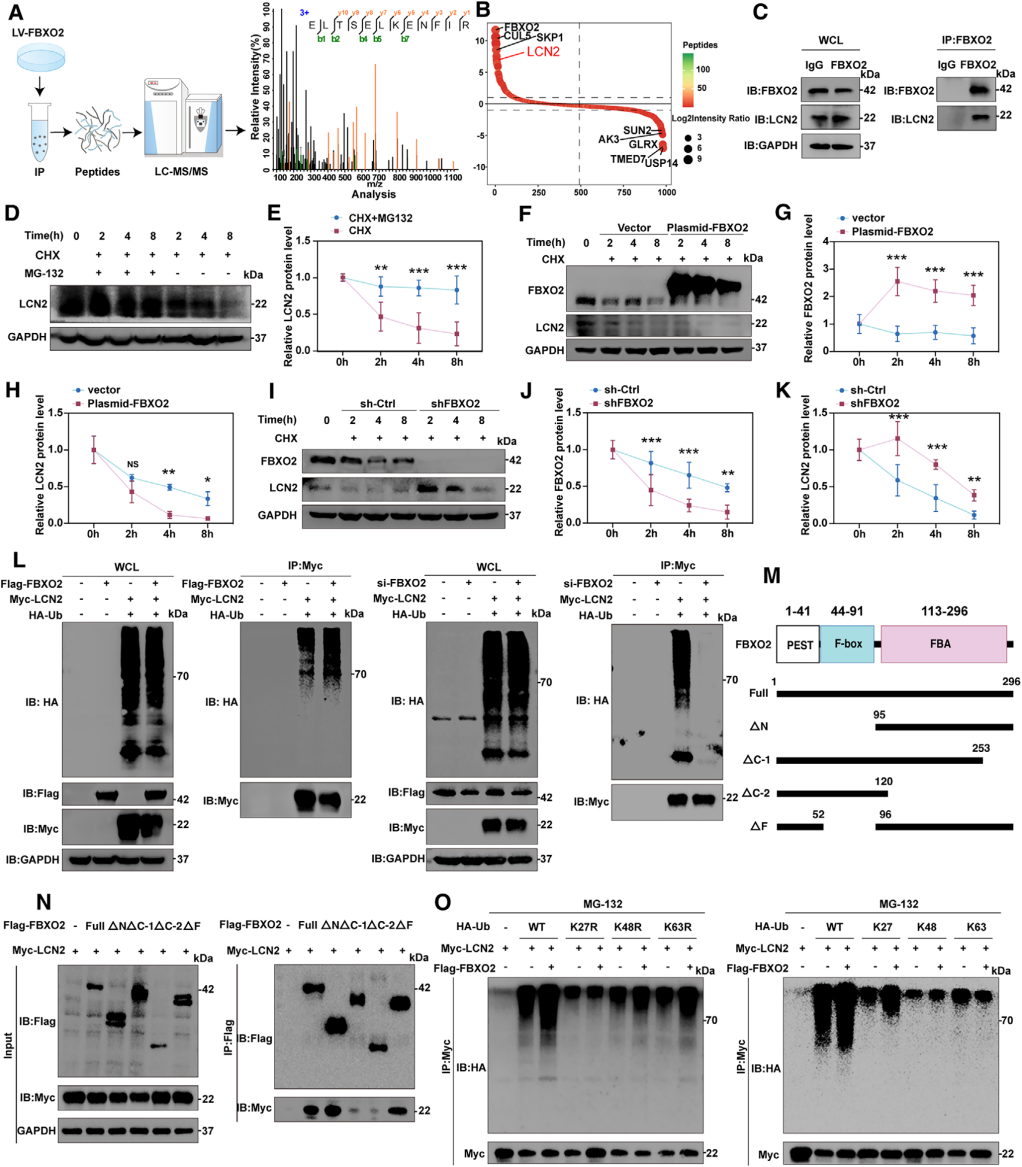
FBXO2 interacted with lipocalin 2 (LCN2) and triggered K27‐linked LCN2 ubiquitination and degradation. A,B) The result of immunoprecipitation‐mass spectrometry (IP‐MS) indicated that LCN2 was identified as a putative target protein of FBXO2. C) Endogenous LCN2 precipitated by FBXO2 in human nucleus pulposus (NP) cells. D,E) The expression of LCN2 detected over time in the presence of the protein synthesis inhibitor cycloheximide (CHX, 10 µg mL^−1^) and the proteasome‐specific inhibitor MG132 (10 × 10^−6^ m) in HEK293T cells. *n* = 3. F–K) The expression of LCN2 detected over time in HEK293T cells after knockdown or overexpression of FBXO2, followed by treatment with CHX. *n* = 3. L) HEK293T cells were co‐transfected with Flag‐FBXO2 or si‐FBXO2, Myc‐LCN2, and HA‐Ub. Western blot (WB) analysis indicated that FBXO2 ubiquitinated LCN2. M) Full‐length or truncated segments of Flag‐FBXO2, containing different domains, transfected into HEK 293T cells to investigate the binding region. N) The Co‐IP revealed that the fragment containing FBA domain of FBXO2 was able to bind LCN2 in HEK293T cells. O) The ubiquitination signals of LCN2 persisted in cells expressing the K48R and K63R mutants, but abolished in cells expressing the K27R mutant. Ubiquitination of LCN2 detected in cells expressing the K27‐only variant, with no detectable signals in cells expressing K48‐only or K63‐only mutants. All data are shown as the mean ± SD. Two‐way repeated‐measures ANOVA with Bonferroni correction (E, G, H, J, and K) was used to determine the statistical significance. * for *P* < 0.05, ** for *P* < 0.01, *** for *P* < 0.001, NS for no significance.

As an E3 ubiquitin ligase, FBXO2 binds to substrate proteins to mediate their biological functions.^[^ ^39^ ^]^ To investigate its regulatory role in oxidative stress‐induced ferroptosis, we performed immunoprecipitation‐mass spectrometry (IP‐MS) in human NP cells. This analysis identified LCN2 as a candidate FBXO2‐interacting protein (**Figure** **6**A,B). LCN2, an innate immune protein, has been identified as playing a pivotal role in iron regulation under both physiological and inflammatory conditions.^[^ ^40^ ^]^ Recent studies show that LCN2 induces ferroptosis in multiple diseases, such as age‐related macular degeneration and lung cancer cachexia.^[^ ^41^, ^42^ ^]^ Therefore, we chose LCN2 for further research. Reciprocal Co‐IP assays further confirmed the result of IP‐MS: endogenous LCN2 was specifically precipitated by FBXO2 but not control IgG in human NP cells (Figure 6C). Further validation in HEK293T cells demonstrated that exogenous Flag‐FBXO2 pulled down endogenous LCN2, while Myc‐LCN2 reciprocally precipitated endogenous FBXO2 (Figure S6H,I, Supporting Information). Direct interaction between ectopically expressed Flag‐FBXO2 and Myc‐LCN2 was also confirmed by co‐precipitation (Figure S6J,K, Supporting Information). To map the binding domain, we generated full‐length and truncated Flag‐FBXO2 constructs (Figure 6M). The fragment containing the FBA domain was identified as essential for LCN2 binding (Figure 6N). Functional studies using siRNA‐mediated knockdown revealed an inverse correlation: FBXO2 overexpression reduced LCN2 protein levels, while FBXO2 depletion increased LCN2 abundance (Figure S6B‐G, Supporting Information). The time‐dependent decrease in LCN2 expression was rescued by proteasomal inhibition with MG132 (Figure 6D,E). Consistently, Flag‐FBXO2 overexpression accelerated LCN2 degradation in a dose‐dependent manner, whereas FBXO2 knockdown stabilized LCN2 (Figure 6F–K). Ubiquitination assays showed that FBXO2 overexpression enhanced LCN2 polyubiquitination while reducing its stability. Conversely, FBXO2 knockdown diminished ubiquitination and increased LCN2 accumulation (Figure 6L).

To determine the specific ubiquitin chain topology mediating LCN2 polyubiquitination, we generated mutant ubiquitin variants through site‐directed mutagenesis in HEK293 cells. Three single‐point mutants were constructed by replacing lysine 27, 48, or 63 with arginine (designated K27R, K48R, K63R). Three additional mutants were engineered to retain only lysine 27 (K27‐only), lysine 48 (K48‐only), or lysine 63 (K63‐only) through systematic substitution of all other lysine residues. Co‐transfection experiments were performed with Myc‐LCN2 and Flag‐FBXO2 expression vectors (Figure S6L,M, Supporting Information). Immunoblot analysis revealed distinct ubiquitination patterns: LCN2 ubiquitination signals persisted in cells expressing K48R and K63R mutants but were abolished in K27R‐expressing cells. Notably, Flag‐FBXO2‐mediated ubiquitination was specifically observed in cells expressing the K27‐only variant, with no detectable signals in K48‐only or K63‐only mutants (Figure 6O). These data demonstrate that FBXO2 specifically promotes K27‐linked polyubiquitination of LCN2, establishing its role as the E3 ligase responsible for LCN2 proteasomal degradation. Furthermore, WB results indicated that LCN2 expression was increased in the severe degeneration group (Grade V) compared to the mild degeneration group (Grade II), consistent with its role as an FBXO2 substrate (Figure S6A, Supporting Information). As mentioned above, FBXO2 was decreased in the severe degeneration group, which strengthens the clinical relevance of the FBXO2‐LCN2 axis.

### 2.6 FBXO2 Regulated LCN2‐Mediated Ferroptosis and Mitochondrial Dysfunction in Human NP Cells during IVDD

**Figure 7:**
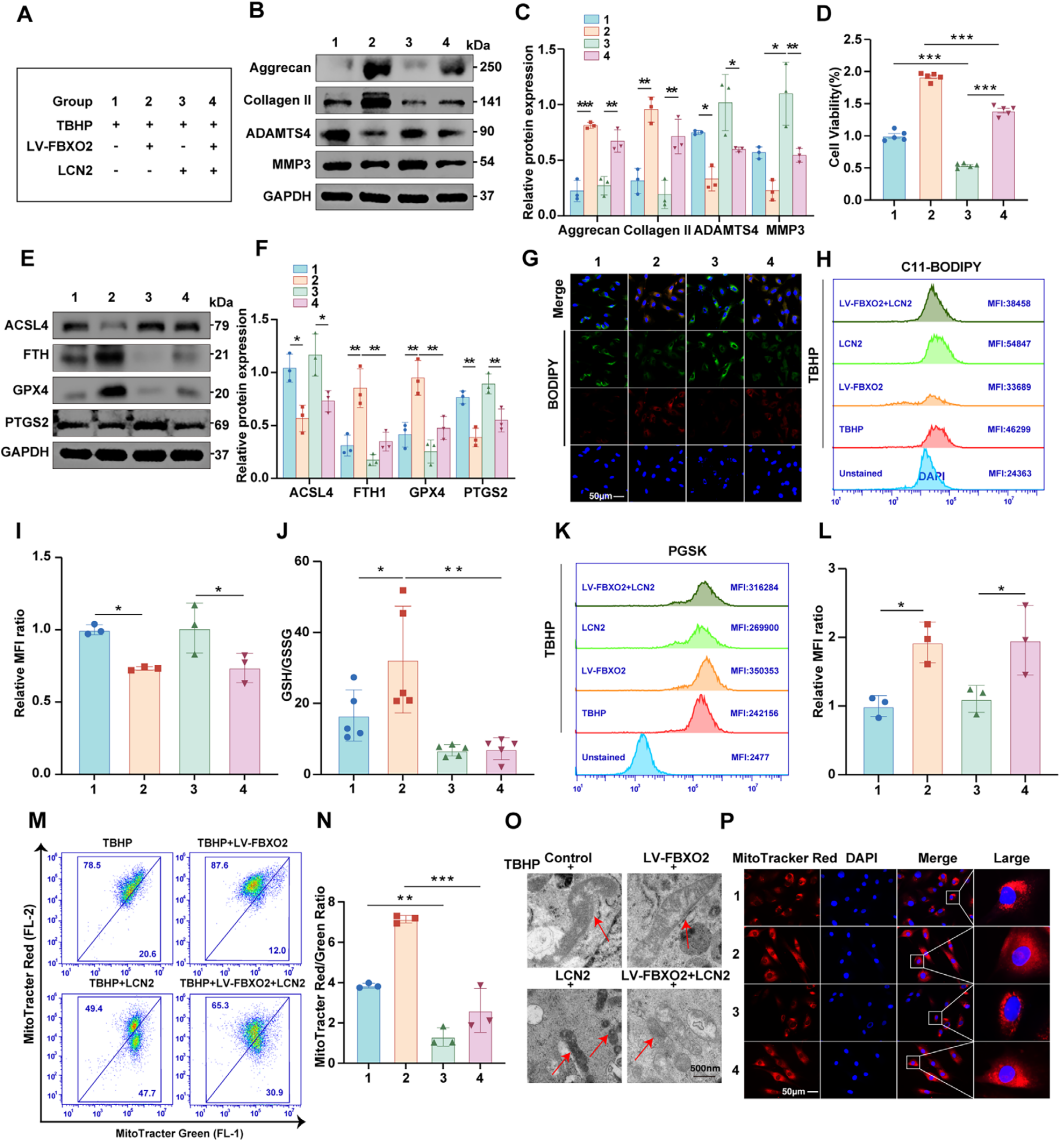
FBXO2 regulated ferroptosis and mitochondrial dysfunction induced by lipocalin 2 (LCN2) in human nucleus pulposus (NP) cells during intervertebral disc degeneration (IVDD). A) Detailed information regarding the experimental group in the relevant part of the experiment. B,C) The protein levels of aggrecan, collagen II, a disintegrin and metalloproteinase with thrombospondin 4 (ADAMTS4), and matrix metallopeptidase 3 (MMP3) detected by Western blot (WB) in human NP cells. *n* = 3. D) Cell viability indicated by the results of the assays. *n* = 5. E,F) In human NP cells, the protein levels of ACSL4, FTH, glutathione peroxidase 4 (GPX4), and PTGS2 assayed by WB. *n* = 3. G–I) Lipid reactive oxygen species (ROS) production assessed using the C11‐BODIPY probe staining method, and also by flow cytometry. *n* = 3. Scale bars: 50 µm. J) The glutathione (GSH) content assayed, and the GSH/glutathione disulfide (GSSG) ratio determined. *n* = 5. K,L) Intracellular ferrous iron levels quantified using the Phen Green SK (PGSK) assay, and the resulting data analyzed by flow cytometry. *n* = 3. M,N) Flow cytometry employed to analyze the staining patterns of MitoTracker Red and MitoTracker Green. The ratio of red to green fluorescence intensity, which serves as an indicator of mitochondrial health, then quantified. *n* = 3. O) Shown representative transmission electron microscope (TEM) images of human NP cells from the specified groups, with red arrows highlighting the mitochondria. *n* = 3. Scale bars: 500 nm. P) Mitochondrial morphology visualized with the MitoTracker Red probe. *n* = 3. Scale bars: 50 µm. All data are shown as the mean ± SD. One‐way analysis of variance (ANOVA) was used followed by Tukey's post hoc test (C,D,F,I,J,L, N) to determine the statistical significance. * for *P* < 0.05, ** for *P* < 0.01, *** for *P* < 0.001, NS for no significance.

To delineate the regulatory function of FBXO2 in ferroptosis during IVDD, human NP cells were co‐transfected with LV‐FBXO2 (or LV‐Ctrl) and plasmid‐LCN2 (or plasmid‐Ctrl) to modulate the expression of FBXO2 and LCN2, respectively (**Figure** **7**A). The WB analysis demonstrated that LCN2 overexpression induced a catabolic shift, characterized by significant upregulation of ADAMTS4 and MMP3, alongside downregulation of the anabolic components aggrecan and collagen II (Figure 7B,C). Notably, LCN2 overexpression exacerbated ferroptosis‐associated molecular signatures, including elevated ACSL4 and PTGS2 levels with concomitant suppression of GPX4 and FTH. These pathological changes were effectively rescued by FBXO2 overexpression (Figure 7E,F).

Functional characterization revealed that LCN2 overexpression markedly reduced cell viability and GSH levels, while increasing lipid peroxidation (BODIPY C11 fluorescence) and intracellular iron accumulation (using PGSK assay). Importantly, LV‐FBXO2 transfection attenuated these deleterious effects (Figure 7D,G–L). Mitochondrial assessment via dual‐parameter flow cytometry (MitoTracker Red/Green fluorescence ratio) and fluorescence imaging revealed severe mitochondrial dysfunction upon LCN2 overexpression (Figure 7M,N,P). TEM further confirmed ultrastructural mitochondrial damage, including cristae fragmentation and vacuolization. Strikingly, FBXO2 overexpression restored mitochondrial integrity, as evidenced by normalized mitochondrial morphology and preserved ultrastructure (Figure 7M–P).

### 2.7 FBXO2 Modulated LCN2‐Mediated Ferroptosis during the Progression of IVDD in Vivo

**Figure 8:**
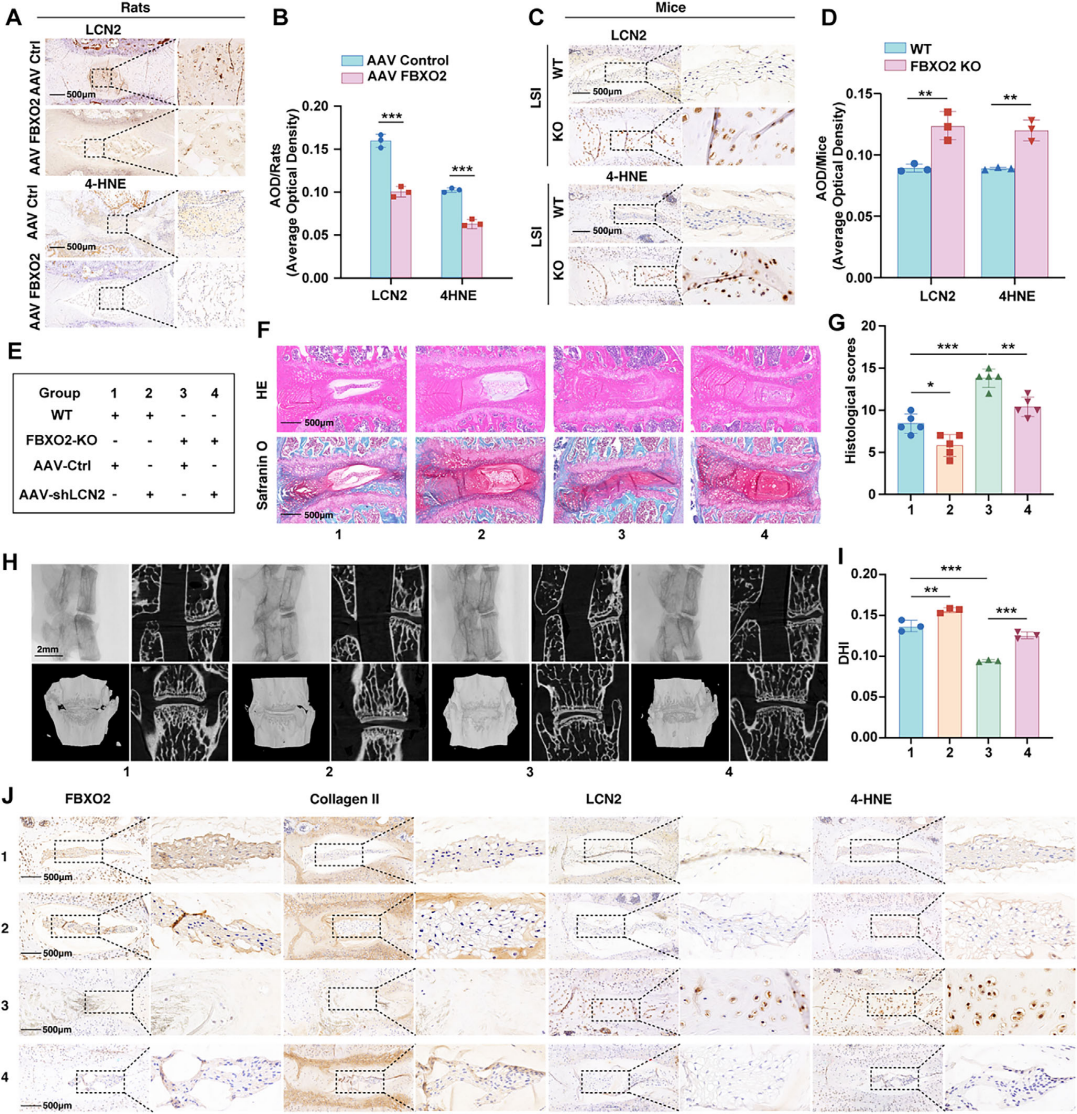
FBXO2 modulated lipocalin 2 (LCN2)‐mediated ferroptosis during the progression of intervertebral disc degeneration (IVDD) in vivo. A,B) The immunohistochemistry (IHC) staining for LCN2 and 4‐hydroxynonenal (4‐HNE) performed on rats with or without FBXO2 overexpression. *n* = 3. Scale bars: 500 µm. C,D) The IHC staining for LCN2 and 4‐HNE performed on wild‐type (WT) and knockout (KO) mice with lumbar spine instability (LSI) model construction. *n* = 3. Scale bars: 500 µm. E) The relevant part of the experiment contains detailed information about the experimental group. F,G) Hematoxylin and eosin (H&E) and Safranin‐O and Fast Green (SO&FG) staining performed in the indicated groups, and histological scores calculated. *n* = 5. Scale bars: 500 µm. H,I) Micro‐computed tomography (Micro‐CT) and reconstruction images of the coronal and sagittal planes of the lumbar spine obtained from the relevant group of mice. Quantification of the micro‐CT analyses performed for disc height index (DHI). *n* = 3. Scale bars: 2 mm. J)The IHC staining for FBXO2, collagen II, LCN2, and 4‐HNE performed in the indicated group. *n* = 3. Scale bars: 500 µm. All data are shown as the mean ± SD. Two‐tailed unpaired Student's *t*‐tests B,D) and one‐way analysis of variance (ANOVA) were used followed by Tukey's post hoc test G,I) to determine the statistical significance. * for *P* < 0.05, ** for *P* < 0.01, *** for *P* < 0.001, NS for no significance.

To dissect the FBXO2‐LCN2‐ferroptosis regulatory axis during IVDD pathogenesis, we analyzed LCN2 expression and the ferroptosis marker 4‐hydroxynonenal (4‐HNE) following FBXO2 overexpression in rats and FBXO2 KO in mice. Results demonstrated that FBXO2 overexpression reduced LCN2 protein levels concomitant with ferroptosis inhibition, whereas FBXO2 KO produced opposing effects (**Figure** **8**A–D). To further validate this regulatory axis, we administered AAV9 vectors encoding LCN2‐specific shRNA or control constructs via intradiscal injection in both WT and FBXO2 KO mice (Figure 8E). Notably, LCN2 knockdown markedly alleviated IVDD severity in FBXO2 KO mice, as reflected by significantly lower histological scores compared to shRNA‐control‐treated KO counterparts (Figure 8F,G). Micro‐computed tomography (Micro‐CT) analyses substantiated these observations, revealing that LCN2 suppression partially restored the diminished disc height index (DHI) percentage induced by FBXO2 KO (Figure 8H,I). Concordant with these findings, IHC staining showed that FBXO2 deficiency‐driven upregulation of LCN2 and 4‐HNE, coupled with collagen II downregulation, were substantially reversed upon LCN2 silencing (Figure 8J and Figure S7A‐D, Supporting Information). These in vivo data collectively demonstrate that FBXO2 regulates IVDD progression via modulation of LCN2‐associated ferroptosis pathways. Our findings further reveal that the exacerbated IVDD phenotype in FBXO2 KO mice is mechanistically attributable to LCN2 activation, highlighting FBXO2's essential role in preserving disc homeostasis through suppression of LCN2‐mediated ferroptotic cascades.

## 3 Discussion

LBP, affecting over 700 million people globally, leads to significant functional limitations and reduced quality of life.^[^ ^1^, ^43^ ^]^ As a major contributor to disability and healthcare consultations, LBP imposes substantial socioeconomic burdens due to absenteeism and medical costs.^[^ ^44^ ^]^ This multifactorial condition involves biological, psychological, and social factors, with IVDD being a key contributor, particularly in aging populations and individuals with spinal injuries.^[^ ^45^, ^46^ ^]^ The intervertebral disc, an avascular structure composed of the NP, AF, and EP, provides compressive and tensile strength to maintain spinal flexibility. However, aging, mechanical stress, and genetic influences drive IVDD progression, characterized by reduced proteoglycan content, disc height loss, endplate hardening, and bone spur formation.^[^ ^47^ ^]^ Recent advances highlight mitochondrial dysfunction—driven by oxidative stress, impaired mitophagy, and ferroptosis—as central to IVDD pathogenesis.^[^ ^48^ ^]^ Our study identifies FBXO2 as a critical molecular hub that integrates mitophagy restoration and ferroptosis suppression in NP cells, offering new mechanistic insights into IVDD progression.

The downregulation of FBXO2 in degenerated NP tissues correlates with IVDD severity, suggesting its role as a stress‐responsive guardian against pathological degeneration. This decline in FBXO2 expression under oxidative stress may stem from KLF10 suppression, which our ChIP data indicate is transcriptionally active in healthy discs but attenuated in degenerated states. KLF10, a stress‐sensitive transcription factor, is likely inhibited by ROS‐mediated epigenetic modifications (e.g., DNA hypermethylation at the FBXO2 promoter) or post‐translational modifications (e.g., oxidation‐induced ubiquitination), thereby reducing its ability to activate FBXO2 transcription.^[^ ^49^ ^]^ Such epigenetic silencing could create a self‐perpetuating cycle where reduced FBXO2 exacerbates mitochondrial dysfunction and ferroptosis, further suppressing KLF10 activity.

Regarding mitochondrial quality control, the observation that FBXO2 promotes PINK1/Parkin‐dependent mitophagy highlights its role as a mitophagy enhancer. While the exact mechanism remains unclear, one plausible explanation is that FBXO2 stabilizes the PINK1/Parkin axis by ubiquitinating and degrading mitophagy inhibitors, such as deubiquitinases (e.g., USP30, MUL1), which are known to antagonize Parkin activity.^[^ ^50^, ^51^ ^]^ Critically, our supplementary experiments revealed that neither FBXO2 overexpression nor knockdown altered BNIP3/NIX expression under oxidative stress conditions (Figure S4M,N: Supporting Information), thereby ruling out receptor‐mediated mitophagy pathways. This finding underscores the specificity of FBXO2's action through the canonical PINK1/Parkin pathway rather than alternative autophagy receptors. Alternatively, FBXO2 may directly modify Parkin or PINK1 to enhance their mitochondrial recruitment or catalytic activity, akin to how SCF E3 ligases regulate substrate specificity in other autophagy contexts. The colocalization of LC3 and TOM20 in FBXO2‐overexpressing cells (Figure 4C,D) supports enhanced autophagosome‐mitochondria interactions under stress. The preferential activation of the PINK1/Parkin pathway aligns with its documented role in oxidative stress response.^[^ ^52^ ^]^ Notably, unlike the mTOR pathway, which primarily integrates nutrient signals,^[^ ^53^ ^]^ or the BNIP3/NIX pathway, which is often hypoxia‐induced,^[^ ^54^ ^]^ the PINK1/Parkin axis is uniquely suited to respond to mitochondrial ROS bursts—a hallmark of IVDD pathogenesis. This pathophysiological relevance explains why FBXO2, as a stress‐responsive E3 ligase, prioritizes this pathway. Nevertheless, we acknowledge mTOR as a high‐priority candidate for follow‐up studies, given its emerging role in disc nutrition sensing and autophagy regulation.^[^ ^55^ ^]^ Future investigations should explore potential crosstalk between FBXO2‐mediated ubiquitination and mTOR‐dependent metabolic reprogramming in NP cells.

Ferroptosis, which was defined by Dixon et al. in 2012, is an iron‐ and ROS‐dependent form of cell death characterized by lipid peroxidation, iron accumulation, ROS formation, as well as glutathione depletion.^[^ ^19^, ^56^ ^]^ It has been established that ferroptosis contributes to numerous degenerative diseases, and increasing evidence suggests its involvement in IVDD through reduced viability and enhanced extracellular matrix degradation of NP cells, AF cells, or EP chondrocytes.^[^ ^21^, ^57^, ^58^, ^59^ ^]^ Although several ferroptosis‐related pathways have been identified in intervertebral disc cells, research remains preliminary. Our identification of LCN2 as an FBXO2 substrate provides a mechanistic link between iron metabolism and ferroptosis in NP cells. LCN2's pro‐ferroptotic role likely stems from its dual function as an iron chaperone and inflammatory mediator: by binding siderophores and ferritin, LCN2 increases labile iron pools,^[^ ^60^ ^]^ thereby driving Fenton reaction‐mediated lipid peroxidation. Additionally, LCN2 may amplify inflammatory signaling (e.g., NF‐κB activation) in degenerated discs, creating a pro‐ferroptotic microenvironment.^[^ ^61^ ^]^ Significantly, the K27‐linked polyubiquitination of LCN2 by FBXO2 represents a paradigm shift from canonical degradation pathways. While K48‐linked ubiquitination typically targets substrates for proteasomal degradation and K63‐linked chains mediate nondegradative processes (e.g., DNA repair, kinase activation),^[^ ^62^, ^63^ ^]^ our data demonstrate that FBXO2 employs this atypical K27 chain topology to direct LCN2 degradation. This aligns with emerging evidence that K27‐linked chains can act as degradation signals in specific contexts (e.g., mitochondrial quality control), yet contrasts with their predominant roles in immune regulation and NF‐κB signaling. In cancer biology, K27 ubiquitination often modulates immune checkpoint proteins or antigen presentation;^[^ ^64^ ^]^ whereas in IVDD, our findings indicate that it serves to terminate iron‐driven ferroptotic signals. Such context‐dependent reprogramming of ubiquitin chain functionality underscores the evolutionary plasticity of the ubiquitin‐proteasome system and highlights FBXO2 as a node integrating iron metabolism with proteostasis. Future studies should investigate whether other E3 ligases exploit K27‐linked ubiquitination for substrate‐specific degradation in metabolic disorders involving iron dyshomeostasis.

Importantly, the interplay between mitochondrial dysfunction and ferroptosis creates a self‐perpetuating cycle in IVDD. Damaged mitochondria release ROS and pro‐inflammatory cytokines (e.g., IL‐1β), which activate LCN2 expression and iron influx, while ferroptosis‐induced lipid peroxidation further impairs mitochondrial membranes. FBXO2 disrupts this vicious cycle by (1) clearing damaged mitochondria via mitophagy and (2) degrading LCN2 to limit iron availability. This dual action explains its potent protective effects in rodent models, where AAV9‐FBXO2 not only restored collagen II expression but also reduced disc inflammation and vascularization. The therapeutic relevance of this strategy is underscored by the failure of single‐pathway interventions in previous IVDD studies, highlighting the need for combinatorial approaches.

In vivo, AAV9‐mediated FBXO2 delivery attenuated disc degeneration in rats by suppressing ferroptosis and restoring collagen II expression, whereas FBXO2 KO accelerated IVDD via LCN2‐dependent pathways. To address concerns about systemic FBXO2 ablation affecting nondisc tissues, we performed comprehensive phenotypic and histological analyses of global FBXO2 knockout mice. In naive 3‐month‐old KO mice without IVDD induction, gross morphology, body weight, and organ weights (brain, heart, liver, lung, kidney) were indistinguishable from wild‐type controls (Figure S3A,B: Supporting Information). Histological examination of major organs in LSI‐operated mice further confirmed the absence of pathological alterations, with H&E staining revealing normal tissue architecture (Figure S3C, Supporting Information). Critically, IHC analysis demonstrated efficient FBXO2 ablation in peripheral tissues without compensatory overexpression (Figure S3C, Supporting Information). These data collectively indicate that the accelerated IVDD phenotype in FBXO2 KO mice stems from disc‐intrinsic mechanisms rather than systemic disturbances. While tissue‐specific conditional knockout models (e.g., Acan‐CreERT2 FBXO2fl/fl) would provide definitive evidence of disc‐autonomous effects, our results support the therapeutic specificity of FBXO2 modulation. To strengthen clinical relevance, validation across diverse IVDD models (e.g., aging, tail suspension, mechanical overload) remains warranted. Additionally, dual FBXO2/LCN2 disc‐specific knockout mice should be developed to conclusively establish that FBXO2 mitigates IVDD by targeting LCN2‐dependent ferroptosis.

In conclusion, this study identifies FBXO2 as a critical regulator of IVDD by coordinately restoring PINK1/Parkin‐mediated mitophagy and suppressing LCN2‐driven ferroptosis. FBXO2 downregulation in degenerated discs correlates with KLF10 transcriptional suppression, forming a pathogenic feedback loop exacerbated by oxidative stress. Mechanistically, FBXO2 promotes mitochondrial quality control through activation of the canonical PINK1/Parkin axis while inducing K27‐linked polyubiquitination and proteasomal degradation of LCN2, thereby limiting iron‐dependent ferroptosis. In vivo validation confirms FBXO2's therapeutic potential without systemic toxicity, though conditional knockout models and mTOR pathway crosstalk warrant further investigation. Collectively, these findings position FBXO2 as a multifunctional guardian of disc homeostasis and a promising therapeutic target for IVDD.

## 4 Experimental Section

### Human Samples

Thirty‐nine NP tissues, originating from patients suffering from lumbar disc degeneration, were gathered. These newly acquired NP samples were subsequently immersed in RNAlater solution (supplied by Invitrogen Corp., Carlsbad, CA, USA) and kept at a temperature of 4 °C overnight, prior to being stored at −80 °C for future use. The human degenerative intervertebral disc samples were divided into four categories (II, III, IV, and V) according to the Pfirrmann grading system.^[^ ^65^ ^]^ The summary of the detailed information pertaining to the patient intervertebral disc samples is provided in Table S1 (Supporting Information). All human tissue procedures were conducted in accordance with the ethical standards of the Declaration of Helsinki (World Medical Association, 2013) and approved by the Ethics Committee of Shanghai East Hospital, Tongji University School of Medicine (Reference number: [2022]113). Written informed consent was obtained from all participants.

### Cell Culture and Transfection

Human NP cells, purchased from ScienCell in Carlsbad, CA, were cultured in human NP cell medium (ScienCell, Carlsbad, CA, USA) at 37 °C with 5% CO2, as previously described.^[^ ^30^ ^]^ To induce oxidative stress and inflammatory responses, human NP cells were treated with tert‐butyl hydroperoxide (TBHP, Cat# 458139, Sigma‐Aldrich, St. Louis, MO, USA) and interleukin‐1β (IL‐1β, Cat# SRP3083, Sigma‐Aldrich), respectively. For mechanistic investigation, human NP cells were administered either: (1) Cyclosporine A (CsA, 10 µM; Selleck Chemicals, Houston, TX; Cat# S2286) to inhibit autophagy, or (2) N‐acetylcysteine (NAC, 5 mM; Selleck Cat# S1623) to neutralize ROS. HEK293T cells were cultured in high‐glycemic DMEM containing 10% fetal bovine serum (FBS; Hyclone, Thermo Scientific) and 1% penicillin/streptomycin at 37 °C and 5% CO2. Lentiviruses were constructed according to the manufacturer's instructions (Genomeditech, Shanghai, China) to stably overexpress or knock down KLF10 and FBXO2 in cells (Table S2, Supporting Information). siRNA targeting FBXO2, Parkin, and LCN2 was acquired from Genomeditech (Shanghai, China); the sequences are provided in Table S3 (Supporting Information). Plasmids such as Flag‐tagged FBXO2 (Flag‐FBXO2), Myc‐tagged LCN2 (Myc‐LCN2), and HA‐tagged Ub (HA‐Ub), as well as mutant HA‐Ub and truncated Flag‐FBXO2, were constructed by Miaolingbio (Wuhan, China). These plasmids were transfected into cells using Lipofectamine 3000 (Invitrogen, Carlsbad, CA, USA) or Lipo8000 reagent (C0533; Beyotime, China).

### RNA Sequencing and Analysis

RNA sequencing was conducted by Shanghai Yingbai Biotechnology Co., Ltd. (Shanghai, China). Initially, total RNA was extracted from human NP cells using Trizol. Subsequently, after assessing the quality and quantity of the RNA samples, the samples were reverse‐transcribed to generate cDNA libraries. After purification, the libraries were quantified using a Qubit 3.0 Fluorometer and validated on an Agilent 2100 Bioanalyzer. Following this, sequencing was performed on an Illumina NovaSeq 6000 platform (Illumina, San Diego, CA, USA). A multiple‐hypothesis test correction was applied to the p‐values, with the threshold determined by controlling the false discovery rate (FDR). Differentially expressed genes (DEGs) with an FDR < 0.05 and an absolute log2 fold change (log2FC) ≥ 1 were analyzed using the DESeq2 R package. Finally, the DEGs were visualized through heatmaps and volcano plots.

### Chromatin Immunoprecipitation (ChIP) and Dual‐Luciferase Reporter Assay

ChIP assays were performed according to established protocols with modifications.^[^ ^66^ ^]^ Briefly, cells were cross‐linked with 1% formaldehyde for 10 min at room temperature. Chromatin was then fragmented by sonication to obtain DNA fragments ranging from 100 to 500 bp. Immunoprecipitation was conducted overnight at 4 °C using 2 µg of anti‐KLF10 antibody (sc‐130408; Santa Cruz Biotechnology, Texas, USA). Subsequently, Protein A/G magnetic beads (SB‐EX00; Share‐Bio, China) were added and incubated for 2 h at 4 °C. Parallel samples using normal rabbit IgG served as negative controls. After purification, the DNA was amplified by PCR using specific primers targeting KLF10‐binding regions.

FBXO2 promoter fragments (‐2000 to +100 bp relative to the TSS) were PCR‐amplified from human genomic DNA and cloned into the XhoI/HindIII restriction sites of the pGL4.10[luc2] luciferase reporter vector (Genomeditech, Shanghai, China; sequence verified by Sanger sequencing). KLF10‐overexpressing plasmids or empty vector controls were co‐transfected with 1 µg of pGL4.10‐promoter constructs into HEK293T cells using Lipofectamine 3000 (Invitrogen; Thermo Fisher Scientific). For normalization, 50 ng of the pRL‐TK Renilla luciferase vector (Promega) was included in all transfections. After 48 h of incubation, cells were lysed and luciferase activity was quantified using the Dual‐Luciferase® Reporter Assay System (E1910; Promega).

### RNA Extraction and Quantitative Reverse Transcription‐PCR (qRT‐PCR)

Total RNA was isolated from NP tissues and cells using Trizol reagent (Takara Biotechnology), adhering strictly to the manufacturer's instructions. Spectrophotometry was employed to determine the RNA concentration. Subsequently, cDNAs were reverse‐transcribed using the TaqMan RT Reagents Kit (Applied Biosystems). Quantitative PCR was conducted using a QuantiTect SYBR Green PCR Kit (Takara) on an Applied Biosystems 7500 Real‐Time PCR System. Expression levels of the GAPDH gene were used as an endogenous control. The 2^‐ΔΔCT method was applied to analyze the relative fold changes in target gene expression. The primer sequences are provided in Table S4 (Supporting Information).

### Western Blot (WB) Analysis

Total protein was extracted from human NP cells or tissues using RIPA lysis buffer supplemented with protease and phosphatase inhibitors (P0013C, P1005; Beyotime, China). Protein concentrations were determined with a BCA Protein Assay Kit (P0011; Beyotime, China) according to the manufacturer's protocol. Equal amounts of protein lysates (20 µg per lane) were separated by sodium dodecyl sulfate‐polyacrylamide gel electrophoresis (SDS‐PAGE) and subsequently transferred onto PVDF membranes (0.45 µm, IPVH00010; Millipore). After blocking with 5% nonfat milk in TBST for 1 h at room temperature, the membranes were incubated overnight at 4 °C with primary antibodies listed in Table S5 (Supporting Information). Following three washes with TBST, membranes were probed with horseradish peroxidase (HRP)‐conjugated secondary antibodies (1:5000 dilution) for 1 h at room temperature. Protein signals were developed using ECL substrate (ECL‐1001, Affinity Biosciences, Cincinnati, OH, USA) and captured using a Tanon 5200 chemiluminescence imaging system (Tanon Science & Technology, Shanghai, China). Quantitative analysis of band intensity was performed using ImageJ software (v1.53k, National Institutes of Health).

### Immunofluorescence (IF) Staining

The cells were washed using PBS and then fixed with 4% paraformaldehyde. The tissue sections underwent deparaffinization in xylene and were hydrated through a series of decreasing ethanol concentrations. Subsequently, both the cells and tissue sections were permeabilized with 0.3% Triton X‐100 sourced from Solarbio (China) and blocked with BSA at room temperature. This was followed by an overnight incubation of the primary antibodies (Table S5, Supporting Information) at 4 °C. Subsequently, the cells were incubated in relevant secondary antibodies in the dark for 1 h and counterstained in DAPI for another 5 min. The fluorescence was visualized using a fluorescence microscope (Leica DMI3000B, Leica Microsystems, Inc).

### Histological and Immunohistochemical Staining

All disc samples were fixed in 4% paraformaldehyde, embedded in paraffin, and sectioned into 5‐µm slices. Human and rodent disc samples were stained with hematoxylin and eosin (H&E) and Safranin‐O and Fast Green (SO&FG). Histological images were acquired using a light microscope. Rat disc histology was scored according to a revised histologic grading system.^[^ ^67^ ^]^ Immunohistochemistry (IHC) was performed on human and rodent NP tissues. Briefly, sections underwent antigen retrieval, blocking, and incubation with primary antibodies (Table S5, Supporting Information) followed by species‐matched secondary antibodies. Images were captured under a light microscope, and average optical density (AOD) was quantified using ImageJ.

### Reactive Oxygen Species (ROS) Production Assay

Rosup serves as a positive inducer of ROS. To detect the cytosolic ROS, human NP cells were loaded with 10 × 10^−6^ m DCFH‐DA (S0033S; Beyotime, China) and incubated at 37 °C for 30 min. For mitochondrial ROS, cells were stained with 5 × 10^−6^ m MitoSOX Red (M36008; Invitrogen, Carlsbad, CA, USA) for 10 min. Fluorescence was visualized by confocal microscopy and quantified via flow cytometry (Beckman Coulter).

### Mitochondrial Membrane Potential Analysis

For the 5,5′,6,6′‐Tetrachloro‐1,1′,3,3′‐tetraethylbenzimidazolylcarbocyanine iodide (JC‐1) assay, Carbonyl cyanide m‐chlorophenyl hydrazone (CCCP) served as a positive control by disrupting H⁺ transport and collapsing the electrochemical gradient across the mitochondrial membrane (C2005; Beyotime, China). For the Tetramethylrhodamine ethyl ester (TMRE) assay, NAC was utilized to eliminate ROS due to its antioxidant properties (C2001S; Beyotime, China). In the case of the JC‐1 assay, cells were incubated with the dye for 20 min at 37 °C, washed twice, and then imaged using fluorescence microscopy. Mitochondrial membrane potential was calculated as the ratio of red to green fluorescence. For the TMRE assay, stained cells were analyzed by both fluorescence microscopy and FACScan flow cytometry.

### Autophagic Flux Analysis

Human NP cells, at 60% confluency, were infected with AdV‐mRFP‐GFP‐LC3 (Hanbio, Shanghai, China) in accordance with the manufacturer's instructions. Following an overnight infection period, the culture medium was replaced. Subsequently, fluorescence was observed using a Leica DMI3000B fluorescence microscope after 48 h of infection.

### MitoTracker Staining Assay

Cells were stained with MitoTracker Red (M7512; Invitrogen, Carlsbad, CA, USA) and MitoTracker Green (40742ES50, Yeasen Biotech, Shanghai, China) according to the manufacturer's protocols. Fluorescence intensity was quantified by flow cytometry (10 000 events per sample). Mitochondrial morphology was analyzed using MitoTracker staining followed by fluorescence microscopy imaging (Leica DMI3000B, Leica Microsystems, Inc).

### Colocalization of Mitochondria and Lysosomes

The cells were stained with MitoTracker Green (C1048; Beyotime, China) and LysoTracker Red (C1046; Beyotime, China), following the manufacturer's instructions. Subsequently, pictures were taken using the fluorescence microscope.

### Cell Growth Assay

Cell viability was detected using the CellTiter‐Lumi Plus Luminescent Cell Viability Assay Kit (C0068; Beyotime, China). In brief, human NP cells were seeded in a 96‐well plate at a density of 5 × 10^3^ cells per well. On the second day after plating, an equal volume of CellTiter‐Lumi Plus Reagent was added to the cell culture medium, and the mixture was then agitated on an orbital shaker for 2 min at room temperature to induce cell lysis. Following an additional 10‐min incubation period at room temperature, a microplate reader was used to measure the absorbance at 450 nm.

### Lipid Peroxidation

Lipid peroxidation was assessed using BODIPY 581/591 C11 (D3861; Invitrogen). This fluorescent probe exhibits a characteristic spectral shift from red (590 nm) to green (510 nm) emission with increasing lipid peroxidation levels. In brief, cells were loaded with 2 µM C11‐BODIPY (581/591) and incubated at 37 °C for 60 min under light‐protected conditions. Following two washes with PBS, cellular fluorescence was either visualized using a fluorescence microscope or quantified through flow cytometry after cell harvest and resuspension in assay buffer.

### Intracellular Glutathione (GSH)/Glutathione Disulfide (GSSG) Ratio Detection

The intracellular GSH was detected using GSH and GSSG Assay Kit (S0053; Beyotime, China). Human NP cells underwent repeated cycles of freezing and thawing to achieve lysis, subsequently being centrifuged to obtain the supernatant. This supernatant was then assayed for GSH and GSSG levels, following the manufacturer's instructions.

### Iron Analysis

Intracellular ferrous iron levels in human NP cells were measured utilizing a Phen Green SK(PGSK) assay (GC40243‐500; Glpbio, USA). In brief, cells were treated as indicated before 20 µmol L^−1^ of PGSK was added and incubated for 10 min. The cells were washed twice with PBS to remove any excess PGSK. Subsequently, the cells were trypsinized and resuspended in PBS supplemented with 5% FBS. Detection of ferrous iron was carried out using flow cytometry.

### Transmission Electron Microscope (TEM)

After undergoing experimental interventions, human NP cells were sequentially fixed with 2.5% glutaraldehyde and 2% osmium tetroxide to preserve their ultrastructural features. Following dehydration in acetone, these cells were sectioned into semi‐thin slices using a Leica EM UC7 ultramicrotome and subsequently stained with toluidine blue. The images were captured using a transmission electron microscope, specifically the Tecnai G2 Spirit model manufactured by FEI (Hillsboro, OR, USA).

### 4D Label‐Free Quantitative Proteomics Analysis

The 4D label‐free quantitative proteomics analysis was conducted by APTBIO Co., Ltd. In this part, protein extraction and digestion were performed using SDT buffer followed by the filter‐aided sample preparation (FASP) method. Protein quantification was conducted using the BCA Protein Assay Kit (P0011; Beyotime, China). Digested peptides were desalted, concentrated, and reconstituted in formic acid. SDS‐PAGE was used to visualize protein bands. Mass spectrometry (MS) analysis was carried out on a timsTOF Pro mass spectrometer coupled with Nanoelute, utilizing a reverse phase trap column and analytical column with a linear gradient elution. The MS raw data were analyzed using MaxQuant software for protein identification and quantitation. Bioinformatic analysis included NCBI BLAST+ and InterProScan searches for homologue sequences, gene ontology (GO) annotation using Blast2GO, and kyoto encyclopedia of genes and genomes (KEGG) annotation and pathway mapping. Enrichment analysis was performed based on the Fisher's exact test, with Benjamini‐Hochberg correction for multiple testing, and only functional categories and pathways with p‐values under 0.05 were considered significant.

### Immunoprecipitation (IP) Coupled with MS

Human NP cells stably expressing either an empty vector or Flag‐FBXO2 were lysed using IP buffer (containing 100 mM NaCl, 20 mM Tris‐HCl at pH 8.0, 0.5 × 10^−3^ m EDTA, and 0.5% (v/v) NP‐40), supplemented with a protease inhibitor cocktail. The lysates were cleared by centrifugation and subsequently incubated with ANTI‐FLAG M2 Affinity Gel (Catalog No. A2220; Millipore, Germany) overnight at 4 °C. After being washed with IP buffer five times, the protein samples were resolved by SDS‐PAGE and stained with Coomassie Blue. The proteins that interacted with FBXO2 were identified by MS, which was conducted by Shanghai Advanced Protein Technologies Co., Ltd.

### Co‐Immunoprecipitation (Co‐IP) Assay

Cells were collected and lysed in IP buffer on ice for 20 min. The lysates were cleared by centrifugation at 12 000 rpm for 15 min. For endogenous Co‐IP, the supernatant was incubated with the appropriate antibody overnight, followed by incubation with Protein A/G PLUS‐Agarose (sc‐2003; Santa Cruz, Texas, USA) for 2 h at 4 °C. For exogenous Co‐IP, the supernatant was incubated with Anti‐Myc‐Tag mAb (Agarose Conjugated, M20012; Abmart, Shanghai, China) or ANTI‐FLAG M2 Affinity Gel overnight. The beads were washed three times with IP buffer, denatured at 100 °C for 8 min, and then separated by 12% SDS‐PAGE for immunoblot analysis.

### Construction of Gene Knockout (KO) Mice

FBXO2‐KO mice (Strain S‐KO‐06397) were purchased from Cyagen Biosciences Inc (Suzhou, China) via CRISPR/Cas9‐mediated conventional knockout. The FBXO2 gene (NCBI Gene ID: 230904; RefSeq: NM_176848; Ensembl: ENSMUSG00000041556) is located on chromosome 4 (GRCm39), spanning six exons with the translation initiation codon (ATG) in exon 1 and termination codon (TGA) in exon 6 (Transcript FBXO2‐201: ENSMUST00000047951). Target design: A 607‐bp coding sequence encompassing exons 2–4 was selected for deletion. Four single‐guide RNAs (sgRNAs) were designed to flank this region:

gRNA‐A1: 5′‐TCTCACTGCACGAGGGTCACAGG‐3′

gRNA‐A2: 5′‐GGAACCAAAGCCATCCTAGGAGG‐3′

gRNA‐B1: 5′‐CATACCCTCTCACTGCACGAGGG‐3′

gRNA‐B2: 5′‐CATTTGTGTCCCTCCTAGGATGG‐3′

The Cas9 nuclease complexed with sgRNAs was co‐injected into C57BL/6J fertilized zygotes using a Piezo‐driven micromanipulator. The resulting pups were genotyped using PCR (Table S6, Supporting Information), followed by sequencing analysis to confirm the knockout.

### Construction of a Rodent Model of IVDD and AAV9 Infection

All experimental procedures involving animals were approved by the Institutional Animal Care and Use Committee of Tongji University (Approval No. TJBB05024101) and performed in compliance with the NIH Guide for the Care and Use of Laboratory Animals and ARRIVE guidelines. All protocols met institutional ethical requirements for humane treatment throughout the study. Animals were randomly assigned to experimental groups using computer‐generated randomization. All histological assessments and quantifications were performed by two independent researchers blinded to group allocation. Statistical analyses were conducted prior to unblinding. The rat model of IVDD was established through annulus fibrosus needle puncture, in accordance with established protocols.^[^ ^68^ ^]^ Briefly, adult male Sprague‐Dawley rats weighing 200±10 g were anesthetized via intraperitoneal injection of 2% (w/v) sodium pentobarbital at a dose of 40 mg kg^−1^ body weight. The (coccygeal 6–7) Co6‐7 intervertebral discs were located using X‐rays and subsequently punctured with a 21‐gauge needle. The needle was advanced through the AF into the NP to a depth of 5 mm, maintaining a perpendicular trajectory relative to the disc plane. After rotating 180° clockwise, the needle was stabilized in situ for 30 s to ensure annular injury. Immediately following puncture, 5 µL of AAV9 suspension containing either an FBXO2 overexpression construct or a scramble negative control (titer: 1×10^13^ VG mL^−1^, validated by (PCR; Genechem, Shanghai, China) were slowly infused into the injured discs at a rate of 0.5 µL min^−1^ using a 27‐gauge Hamilton syringe with a 10 µL capacity (Gaoge, Shanghai, China). At 21 days post‐AAV9 injection, D‐luciferin potassium salt (150 mg kg^−1^; Abcam, Cambridge, UK) was administered via intradiscal injection. After a 10‐min incubation period in darkness, bioluminescence signals were captured using an In Vivo Imaging System (IVIS) Spectrum imaging system (PerkinElmer, Waltham, MA, USA). To establish a mouse model of IVDD induced by lumbar spine instability (LSI), eight‐week‐old male mice were anesthetized intraperitoneally with tribromoethanol (T48402; Sigma‐Aldrich) at a dose of 4 mg per 10 g of body weight. Subsequently, the superior and inferior articular processes, supraspinous ligament, and interspinous ligament of the L4‐L5 lumbar vertebrae were removed to induce LSI, which in turn would lead to the development of IVDD. Immediately following the induction of LSI, 0.5 µL of AAV9 expressing LCN2‐specific shRNA (titer: 1×10^12^ VG mL^−1^; Genomeditech, Shanghai, China; Table S7, Supporting Information) was injected intradiscally using a 31‐gauge Hamilton syringe with a 5 µL capacity (Gaoge, Shanghai, China). The mice were then placed in a temperature‐controlled, warm environment after the surgical procedure.

### Micro‐Computed Tomography (Micro‐CT) and Magnetic Resonance Imaging (MRI) Examination

Mice were euthanized, and subsequently, their whole lumbar spine tissues were dissected. After dissection, these tissues were fixed in 4% PFA for a duration of 24–48 h. Following fixation, the lumbar spine tissues underwent examination using a high‐resolution µCT scanner (Bruker SkyScan 1272). The images, captured at a resolution of 2.0 µm per pixel, were then reconstructed utilizing the NRecon v1.7 software. Subsequently, the samples were visualized through the use of CTvox v3.3 software (Bruker Skyscan, Belgium). Additionally, the disc height index (DHI) was calculated for further analysis.The MRI examination was conducted on rats utilizing a 3.0 Tesla MRI system sourced from Philips Eclipse, located in Aachen, Germany. The specific parameters employed during the MRI scanning process were based on those outlined in a previous study.^[^ ^69^ ^]^ Subsequently, the signal intensities of the rat vertebral discs within the sagittal T2‐weighted images were classified according to the Pfirrmann classification system.

### Statistical Analysis

Statistical analyses were conducted using GraphPad Prism 9 software (GraphPad Software, San Diego, CA, USA). All data are presented as means ± standard deviations (SD). The normality of the data was evaluated using the Shapiro‐Wilk test and Kolmogorov‐Smirnov test. The homogeneity of variances was assessed via the Levene test. Statistical comparisons employed an unpaired two‐tailed Student's *t*‐test for dual‐group analyses, one‐way ANOVA with Tukey's post hoc testing for multigroup independent samples, and two‐way repeated‐measures ANOVA with Bonferroni correction specifically applied to longitudinal LCN2 degradation kinetics. Pearson correlation analysis and Spearman's rank‐order correlation were used to evaluate the associations of FBXO2 expression with KLF10 expression levels and Pfirrmann grading scores. Significance levels are denoted as follows: *P < 0.05, **P < 0.01, *** *P* < 0.001, and **** *P* < 0.0001. NS for no significance. Sample sizes (n) for all experiments were ≥3.

## Conflict of Interest

The authors declare no conflict of interest.

## Author Contributions

T.W., Y.W., and B.S. contributed equally to this work. T.W.: Conceptualization, Methodology, Validation, Formal analysis, Data curation, Writing – Original Draft, Visualization, Project Administration. Y.W.: Investigation, Software, Resources, Validation, Writing – Review & Editing, Funding acquisition. B.S.: Methodology, Investigation, Visualization, Formal analysis, Writing – Review & Editing, Data curation. K.G.: Resources, Software, Validation, Investigation, Methodology (specific technique). Z.Z.: Formal analysis, Visualization, Data curation, Software, Validation. Y.L.: Data curation, Validation, Supervision, Writing – Review & Editing. J.Z.: Resources, Investigation (specific experiments), Writing – Review & Editing. D.W.: Conceptualization, Supervision, Funding acquisition, Project administration, Writing – Review & Editing, Guarantor of integrity for entire study.

## Supporting information



Supporting Information

## Data Availability

The data that support the findings of this study are available on request from the corresponding author. The data are not publicly available due to privacy or ethical restrictions.
