# Supplementary material for: FBXO2 Alleviates Intervertebral Disc Degeneration via Dual Mechanisms: Activating PINK1‐Parkin Mitophagy and Ubiquitinating LCN2 to Suppress Ferroptosis
Source: Adv Sci (Weinh). 2025 Aug 12;12(36):e06150. doi: 10.1002/advs.202506150 (PMC12462960; doi:10.1002/advs.202506150)
Supplement: Supplementary file 1 — Supporting Information [file ADVS-12-e06150-s001.docx]

Supporting Information

**FBXO2 Alleviates Intervertebral Disc Degeneration via Dual Mechanisms: Activating PINK1-Parkin Mitophagy and Ubiquitinating LCN2 to Suppress Ferroptosis**

*Tongde Wu, Yanjin Wang, Beiduo Shen, Kai Guo, Ziqi Zhu, Yongzhou Liang, Jianhua Zeng,^*^ and Desheng Wu^*^*

T Wu, B Shen, K Guo, Z Zhu, J Zeng, D Wu.

Department of Spine Surgery, Shanghai East Hospital, School of Medicine, Tongji University, Shanghai 200092, China.

Email: [eastspinewudesheng@126.com](mailto:eastspinewudesheng@126.com); [zengjianhua0411@163.com](mailto:zengjianhua0411@163.com)

T Wu.

Department of Clinic of Spine Center, Xinhua Hospital, School of Medicine, Shanghai Jiao Tong University, 1665 Kongjiang Road, 200092 Shanghai, China.

Y Wang.

Department of Nephrology, Shanghai East Hospital, School of Medicine, Tongji University, Shanghai 200092, China.

Y Liang.

Department of Pediatric Cardiology, West China Second University Hospital, Sichuan University, Chengdu, Sichuan, 610041, China.

Department of Pediatric Cardiology, Xinhua Hospital, School of Medicine, Shanghai Jiao Tong University, 1665 Kongjiang Road, 200092 Shanghai, China.


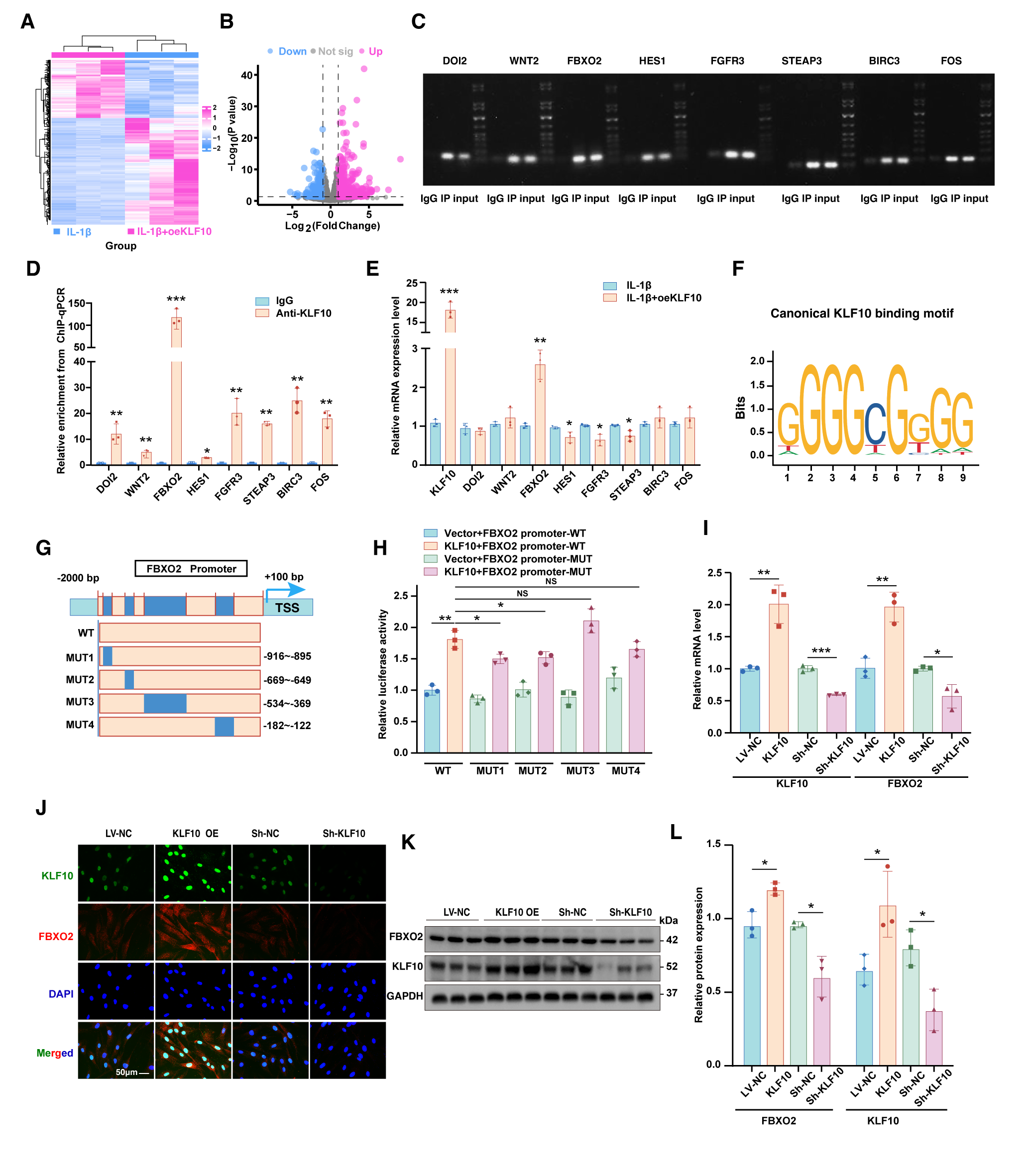
**Figure S1.** Kruppel-like factor 10 (KLF10) transcriptionally activated F-box only protein 2 (FBXO2) expression.

(A, B) RNA sequencing analysis of KLF10-overexpressing human nucleus pulposus (NP) cells identifies differentially expressed genes compared to relevant control. (C) Chromatin immunoprecipitation (ChIP) assay screening revealed eight candidate KLF10 targets. (D) Enrichment score of DOI2, WNT2, FBXO2, HES1, FGFR3, STEAP3, BIRC3, and FOS detected by chromatin immunoprecipitation-quantitative real-time PCR (ChIP-qPCR). (E) mRNA level of KLF10, DOI2, WNT2, FBXO2, HES1, FGFR3, STEAP3, BIRC3, and FOS detected by qPCR. (F, G) JASPER database predicted four KLF10 binding sites in the FBXO2 promoter. (H) Dual-luciferase reporter assay demonstrated that mutations in sites 1 and 2 abolish FBXO2 promoter activity. (I) Expression level of KLF10 and FBXO2 detected by qPCR. (J) Immunofluorescence (IF) staining showed nuclear localization of KLF10 and cytoplasmic/nuclear distribution of FBXO2. Scale bars: 50 μm. (K, L) Expression level of KLF10 and FBXO2 detected by Western blot (WB). All data are shown as the mean ± SD. Two-tailed unpaired Student's t-tests (D and E) and one-way analysis of variance (ANOVA) were used followed by Tukey's post hoc test (H, I, and L) to determine the statistical significance. * for
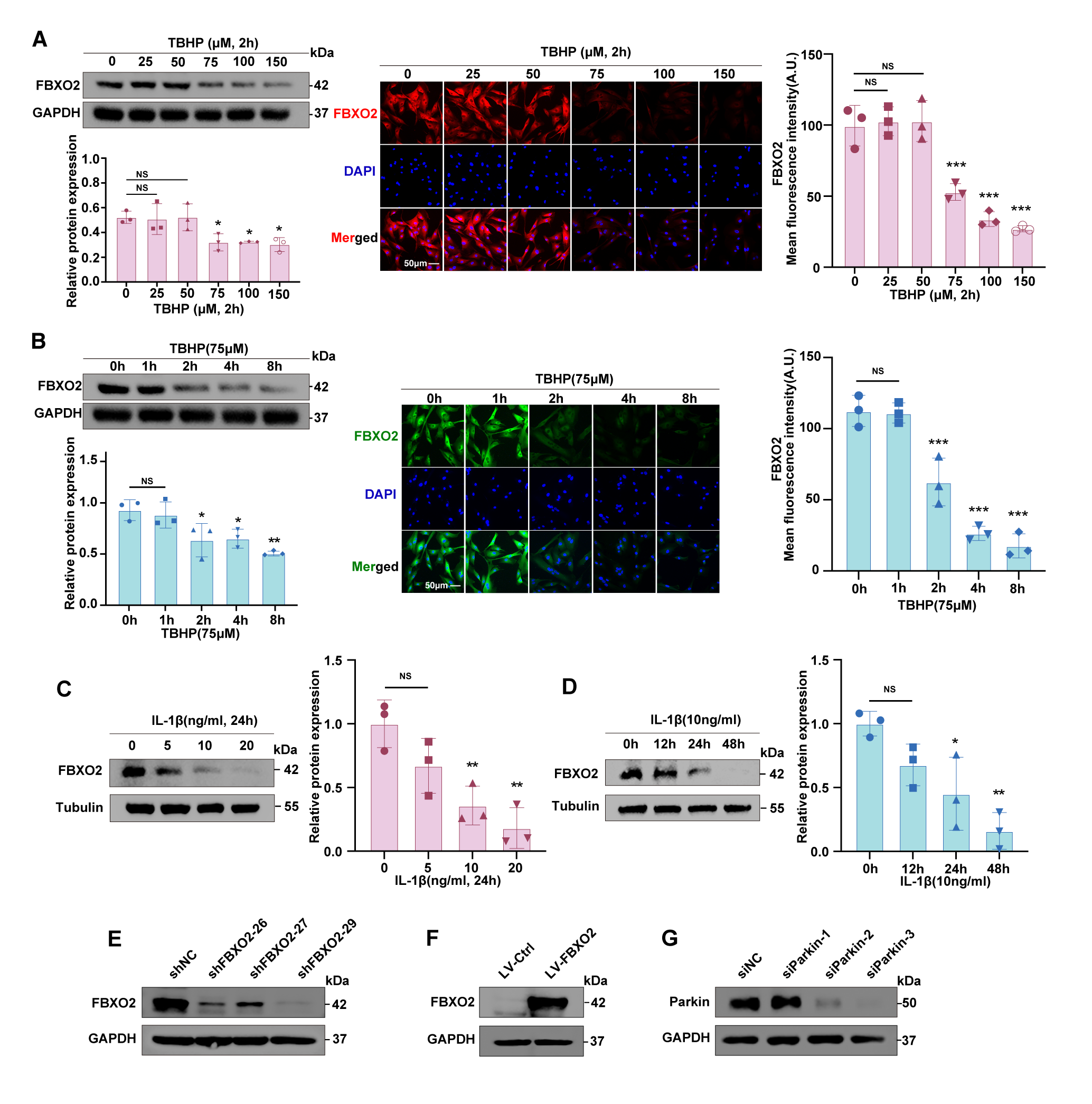
*P* < 0.05, ** for *P* < 0.01, *** for *P* < 0.001, NS for no significance. n=3.

**Figure S2**. Tert-butyl hydroperoxide (TBHP) and interleukin-1β (IL-1β) effectively suppressed FBXO2, thereby modeling disc degeneration in vitro.

(A, B) Under TBHP stimulation, the expression level of FBXO2 assessed via Western blot (WB) and immunofluorescence (IF) staining, with subsequent quantitative statistical analysis. n=3. Scale bars: 50 μm. (C, D) Following IL-1β stimulation, the protein level of FBXO2 measured using WB, followed by quantitative statistical analysis. (E) The protein level of FBXO2 detected by WB after knockdown by shRNA. According to the WB results, shFBXO2-26 chosen for further research. (F) FBXO2 overexpressed through Lentivirus transfection. (G) The protein level of Parkin knocked down by siRNA transfection. siParkin-2 chosen for later use based on WB results. All data are shown as the mean ± SD. Two-tailed unpaired Student's t-tests (A-D) to determine the statistical significance.* for *P* < 0.05, ** for *P* < 0.01, *** for *P* < 0.001, NS for no significance. n=3.


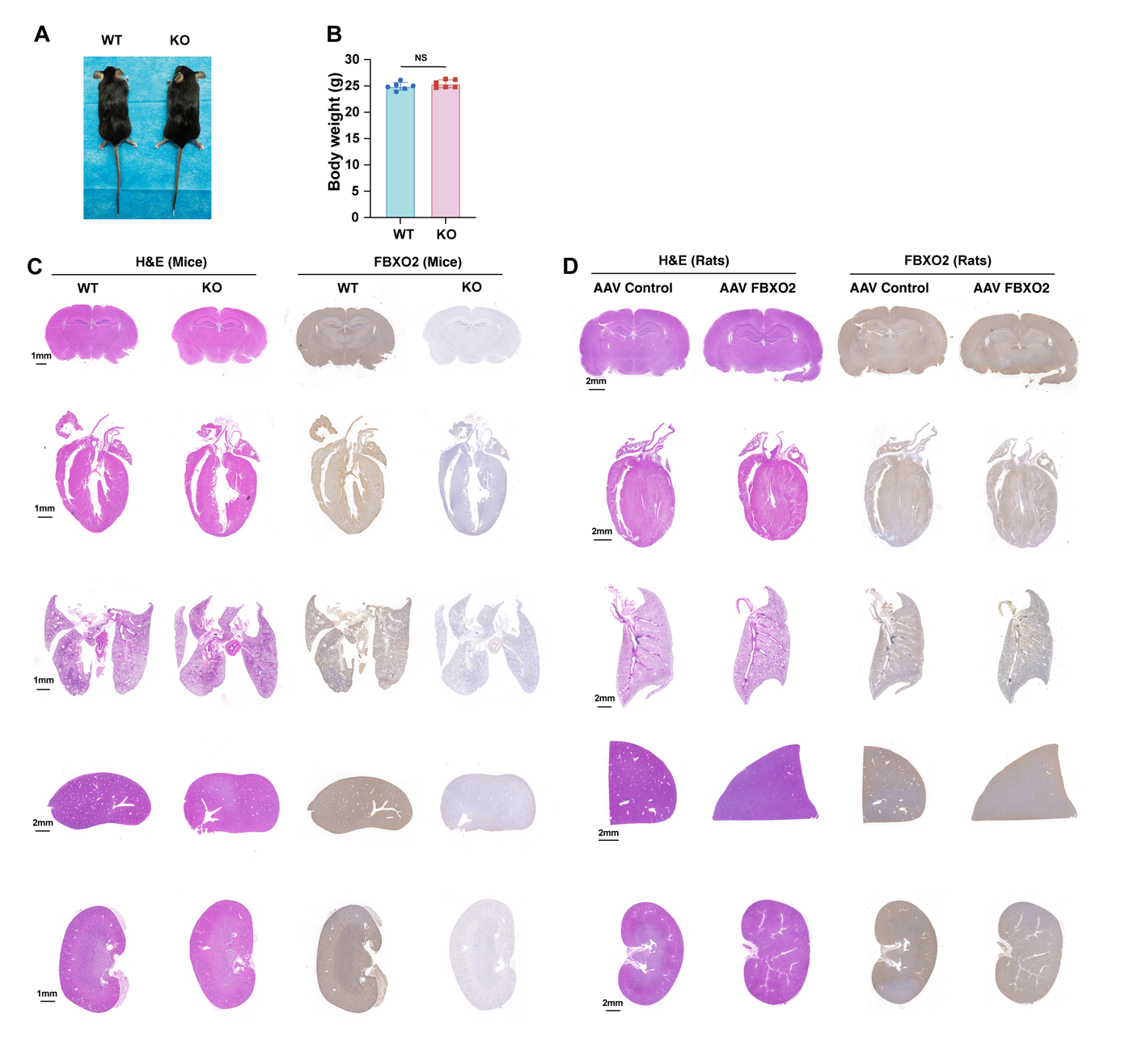
**Figure S3.** Hematoxylin and eosin (H&E) and immunohistochemistry (IHC) staining were performed for FBXO2 in brain, heart, lung, liver, and kidney tissues of mice and rats with the intervertebral disc degeneration (IVDD) model.

(A) FBXO2 wild-type (WT) and knockout (KO) mice. (B) Body weight comparison between WT and KO mice at 3 months of age. n=6. (C) Histological evaluation via H&E and IHC staining for FBXO2 in mouse tissues: Liver tissue. Scale bar: 2 mm. Cross-organ analysis (brain, heart, lung, kidney). Scale bars: 1 mm. (D) Histopathological characterization of rat IVDD models using H&E and IHC (FBXO2) staining. Scale bars: 2 mm. All data are shown as the mean ± SD. Two-tailed unpaired Student's t-tests (B) to determine the statistical significance. NS for no
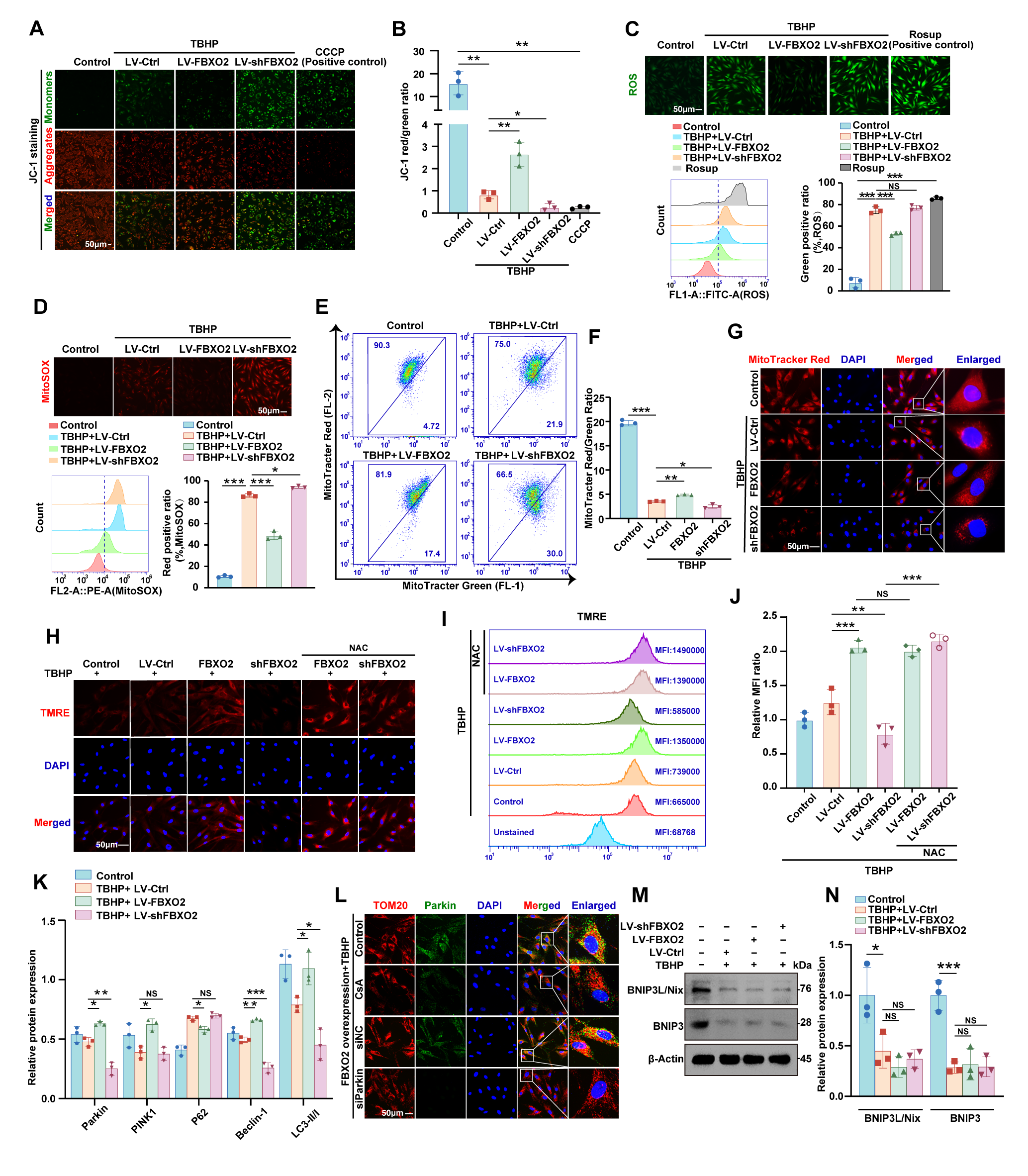
significance.

**Figure S4.** FBXO2 attenuated tert-butyl hydroperoxide (TBHP)-induced mitochondrial oxidative stress in nucleus pulposus (NP) cells by restoring membrane potential, reducing reactive oxygen species (ROS), and improving morphological integrity.

(A, B) The mitochondrial membrane potential detected and quantified by 5,5',6,6'-Tetrachloro-1,1',3,3'-tetraethylbenzimidazolylcarbocyanine iodide (JC-1) assays. Carbonyl cyanide m-chlorophenyl hydrazone (CCCP) served as a positive control, disrupting H⁺ transport and collapsing the electrochemical gradient across the mitochondrial membrane. Scale bars: 50 μm. (C, D) Intracellular ROS levels and MitoSOX levels detected using ROS and MitoSOX assays, respectively, and quantified by flow cytometry. Rosup serves as a positive inducer of ROS. Scale bars: 50 μm. (E, F) MitoTracker Red and MitoTracker Green staining analyzed using flow cytometry. Quantification of the red/green fluorescence intensity ratio performed to assess healthy mitochondrial abundance. (G) Mitochondrial morphology visualized using the MitoTracker Red probe. Scale bars: 50 μm. (H-J) Measurement of mitochondrial membrane potential conducted using the Tetramethylrhodamine Ethyl Ester (TMRE) assay, viewed under a fluorescence microscope, and quantified by flow cytometry. N-acetylcysteine (NAC) utilized to eliminate ROS through its antioxidant properties. Scale bars: 50 μm. (K) The protein levels of PTEN-induced putative kinase 1 (PINK1), Parkin, p62, Beclin-1, and the LC3-II-to-LC3-I ratio quantified. (L) The immunofluorescence (IF) showed co-localization of Parkin and TOM20. Scale bars: 50 μm. (M, N) The protein levels of BNIP3L/Nix and BNIP3 quantified by WB analysis. All data are shown as the mean ± SD. One-way analysis of variance (ANOVA) was used followed by Tukey's post hoc test (B, C, D, F, J, K, and N) to determine the statistical significance. * for *P* < 0.05, ** for *P* < 0.01, *** for *P* < 0.001, NS for no significance. n=3.


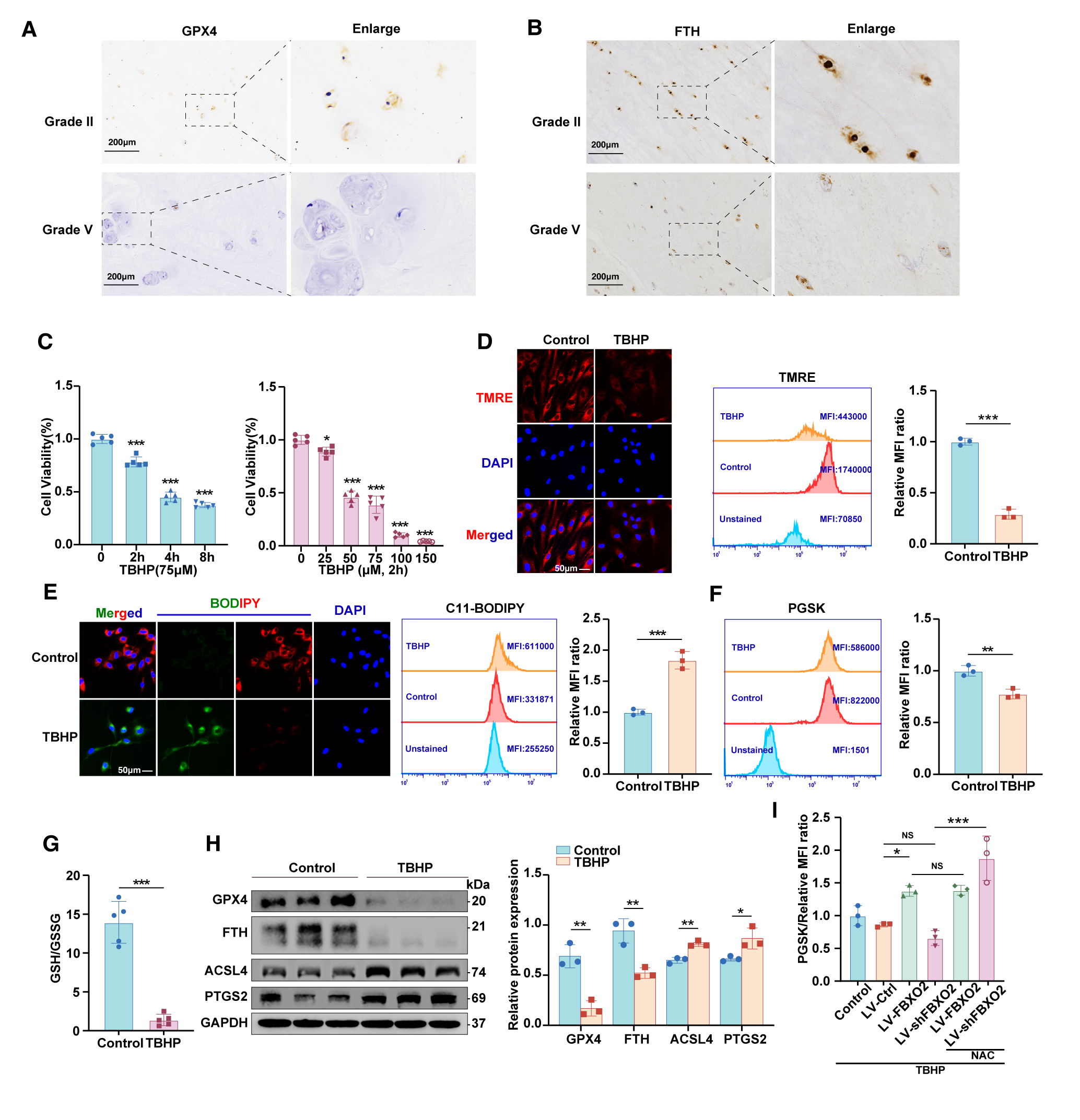


**Figure S5.** Ferroptosis was associated with intervertebral disc degeneration (IVDD), and oxidative stress exacerbated ferroptosis in human nucleus pulposus (NP) cells.

(A, B) The immunohistochemistry (IHC) staining of glutathione peroxidase 4 (GPX4) and FTH in human NP tissues derived from Grade II and Grade V discs. n=3. Scale bars: 200 μm. (C) Cell viability detected by using the cell viability assay. n=5. (D) Assessment of mitochondrial membrane potential by Tetramethylrhodamine Ethyl Ester (TMRE) assay and viewed by fluorescence microscope. n=3. Scale bars: 50 μm. (E) Lipid reactive oxygen species (ROS) production measured using the C11-BODIPY assay. n=3. Scale bars: 50 μm. (F) Intracellular ferrous iron levels measured using the Phen Green SK (PGSK) assay, and the results assessed by flow cytometry. n=3. (G) The glutathione (GSH) content detected by its assay, and the GSH/glutathione disulfide (GSSG) ratio calculated. n=5. (H) The protein levels of GPX4, FTH, ACSL4, and PTGS2 detected by Western blot (WB) in human NP cells. n=3. (I) Intracellular ferrous iron levels measured using the PGSK assay and then quantified. n=3. All data are shown as the mean ± SD. Two-tailed unpaired Student's t-tests (C-H) and one-way analysis of variance (ANOVA) were used followed by Tukey's post hoc test (I) to determine the statistical significance. * for *P* < 0.05, ** for *P* < 0.01, *** for *P* < 0.001, NS
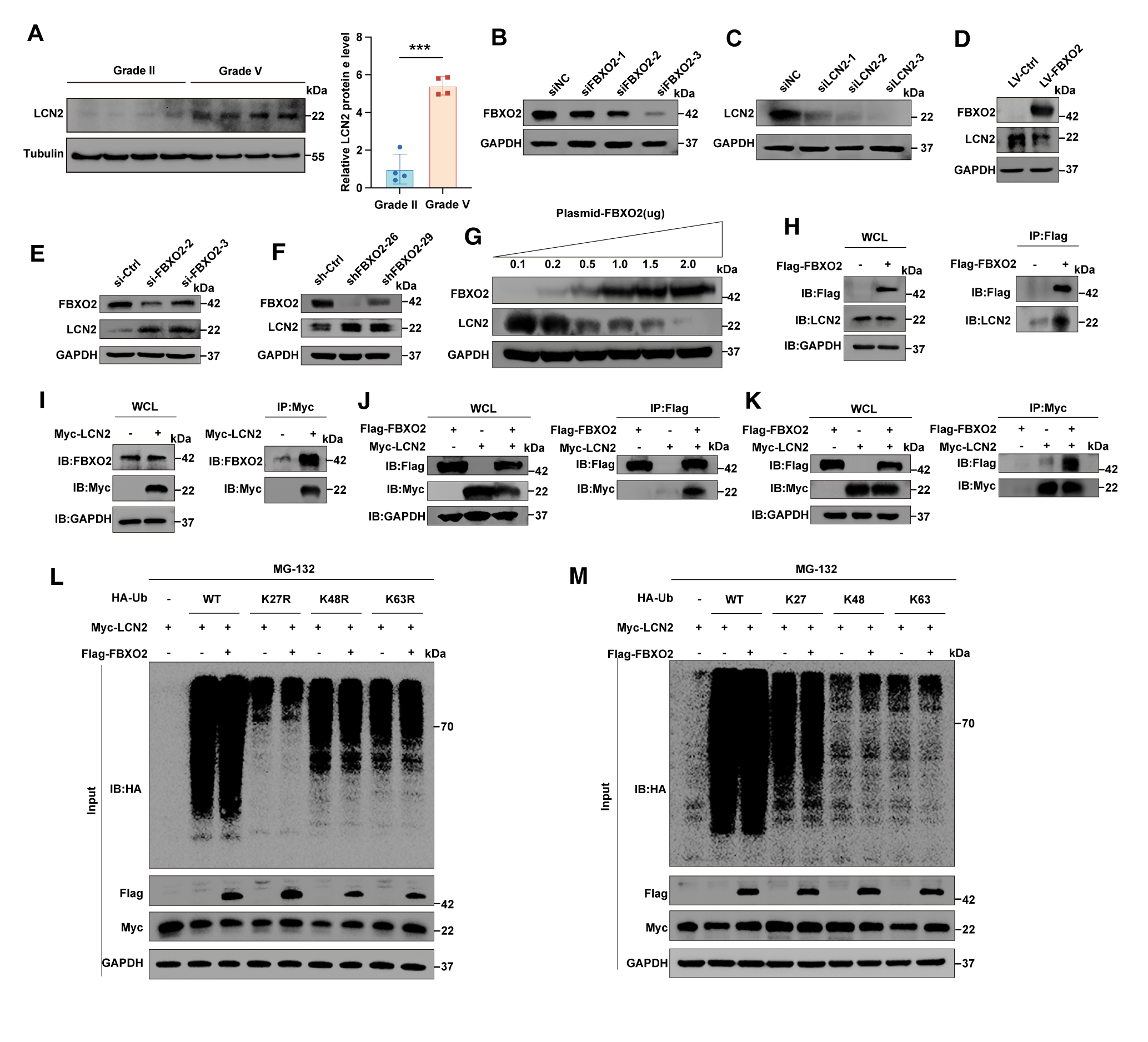
for no significance.

**Figure S6.** FBXO2 interacted with lipocalin 2 (LCN2) and triggered K27-linked LCN2 ubiquitination and degradation.

(A) The protein levels of LCN2 detected by Western blot (WB) in human nucleus pulposus (NP) tissues from Grade II and Grade V discs. n=4. (B, C) The protein levels of FBXO2 and LCN2 detected by WB after they had been knocked down by siRNA. (D-F) The levels of the proteins LCN2 and FBXO2 measured after overexpression or knockdown of FBXO2 in HEK293T cells. (G) FBXO2 overexpression accelerated LCN2 degradation in a dose-dependent manner in HEK293T cells. (H-K) Co-immunoprecipitation (Co-IP) confirmed FBXO2-LCN2 interaction in HEK293T cells. (L, M) The plasmids of Flag-FBXO2, Myc-LCN2, and HA-Ub (including variants K27R, K48R, K63R, K27-only, K48-only, and K63-only) co-transfected into HEK 293T cells that were treated with MG132. All data are shown as the mean ± SD. Two-tailed unpaired Student's t-tests (A) to determine the statistical significance. * for *P* < 0.05, ** for *P* < 0.01, *** for *P* < 0.001, NS for no significance.


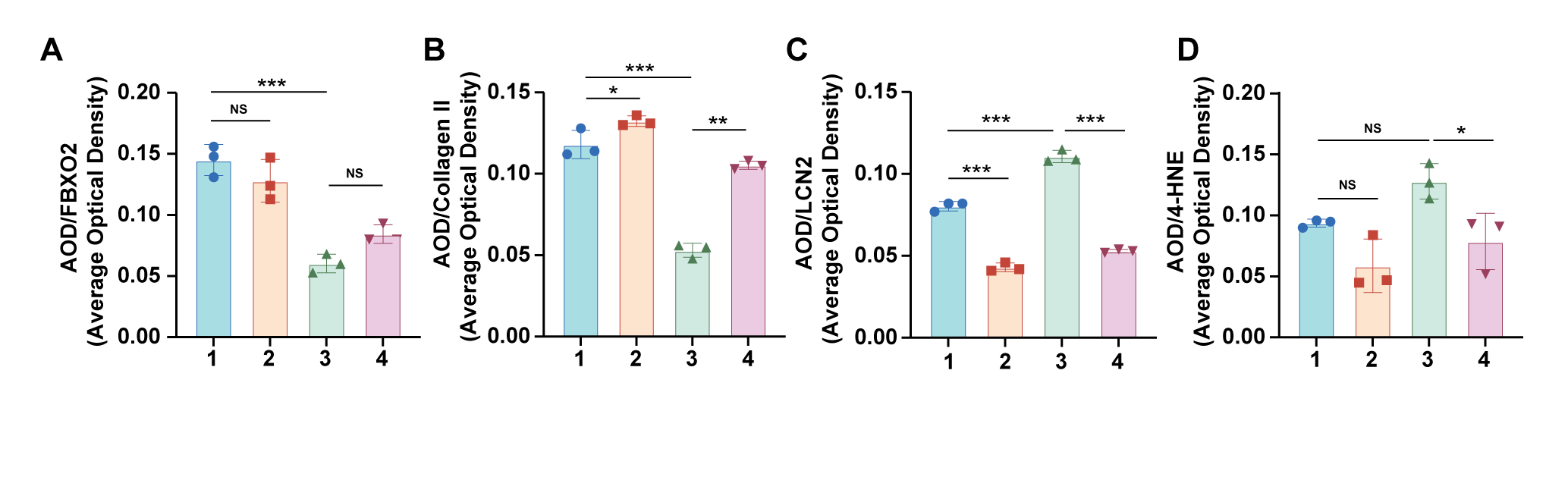
**Figure S7.** Inhibition of lipocalin 2 (LCN2) ameliorated intervertebral disc degeneration (IVDD) induced by knockout (KO) of FBXO2 in mice.

(A-D) The average optical density (AOD) calculated based on the immunohistochemistry (IHC) staining of FBXO2, collagen II, LCN2, and 4-hydroxynonenal (4-HNE) that was performed in the indicated group. All data are shown as the mean ± SD. One-way analysis of variance (ANOVA) was used followed by Tukey's post hoc test (A-D) to determine the statistical significance. * for *P* < 0.05, ** for *P* < 0.01, *** for *P* < 0.001, NS for no significance. n=3.

**Table S1.** Clinical information of human degenerative disc samples from 39 patients

**Table S2.** Sequences of Kruppel-like factor 10 (KLF10) and FBXO2 shRNA

| **KLF10** | |
| --- | --- |
| shNC | TTCTCCGAACGTGTCACGT |
| shRNA1 | GCAAGAAAGAACATACCATGT |
| shRNA2 | GGAGTGACCATTTGACCAAGC |
| shRNA3 | GCTAAATGACATTGCTCTACC |
| **FBXO2** | |
| shNC | TTCTCCGAACGTGTCACGT |
| shRNA1 | ATGAGAGCGTCAAGAAGTACT |
| shRNA2 | TCGCAAAGCACAGGTCATTGA |
| shRNA3 | AGCAGTTCTACTTCCTGAGCA |

| **Variables** | **Number** |
| --- | --- |
| **Sex** | |
| Male  Female | 23  16 |
| **Age** | |
| <50  ≥50 | 11  28 |
| **Diagnosis** | |
| Lumbar disc herniation  Lumbar spinal stenosis | 20  19 |
| **Level**  L1/2 1  L2/3 3  L3/4 6  L4/5 16  L5/S1 13  **Degenerative grades**  II 7  III 19  IV 7  V 6 | |

**Table S3.** Sequences of siRNA

| **FBXO2** | **Sequence (5'-3')** |
| --- | --- |
| FBXO2(h)-si-1 | CUUUGAGUGGUGUCGCAAA tt  UUUGCGACACCACUCAAAG tt |
| FBXO2(h)-si-2 | GAGGGUAGAUAGGCCUUAA tt  UUAAGGCCUAUCUACCCUC tt |
| FBXO2(h)-si-3 | ACAAAGUGGGCUCUCAAUAAA tt  UUUAUUGAGAGCCCACUUUGU tt |
| **Parkin** | **Sequence (5'-3')** |
| Parkin(h)-si-1 | CCAGCAUCUUCCAGCUCAA TT  UUGAGCUGGAAGAUGCUGG TT |
| Parkin(h)-si-2 | GCUUAGACUGUUUCCACUU TT  AAGUGGAAACAGUCUAAGC TT |
| Parkin(h)-si-3 | UGCAGUGCCGUAUUUGAAG TT  CUUCAAAUACGGCACUGCA TT |
| **LCN2** | **Sequence (5'-3')** |
| LCN2(h)-si-1 | GAACUUCCAGGACAACCAA (dT)(dT)  UUGGUUGUCCUGGAAGUUC (dT)(dT) |
| LCN2(h)-si-2 | CCACCAUCUAUGAGCUGAA (dT)(dT)  UUCAGCUCAUAGAUGGUGG (dT)(dT) |
| LCN2(h)-si-3 | GGAGCUGACUUCGGAACUA (dT)(dT)  UAGUUCCGAAGUCAGCUCC (dT)(dT) |

**Table S4.** Sequences of primers

| **Primer Name** | **Sequence (5'-3')** |
| --- | --- |
| KLF10 | F: ATTTGCGGCCGCGATGGTGGGTGA  R: GGGGGCCCATCCTAAAACACGTAA |
| DIO2 | F: AAGGAGGTGACAACAGTGGCAATG  R: GAGCCTCATCAATGTAGACCAGCAG |
| WNT2 | F: TCTCGGTGGAATCTGGCTCTGG  R: CGGAACTGGTGCTGGCATTCTG |
| FBXO2 | F: GACTTGGAAGGCTGGTGTGACG  R: TAGTCGGTGAAGGTGTGGGAGATC |
| HES1 | F: GACCCAGATCAATGCCATGACCTAC  R: AACACCTTAGCCGCCTCTCCAG |
| FGFR3 | F: CGCTAACACCACCGACAAGGAG  R: CACCACCAGGATGAACAGGAAGAA |
| STEAP3 | F: GCTCTTCGTCTGCTTCTATGCCTAC  R: CGATCTGCTTGCGGTGCTGTAG |
| BIRC3 | F: TTGCTTTGCCTGTGGTGGAAAATTG  R: TGGCTTGAACTTGACGGATGAACTC |
| FOS | F: CAAGCGGAGACAGACCAACTAGAA  R: ATTGAGGAGAGGCAGGGTGAAGG |
| GAPDH | F: TGACATCAAGAAGGTGGTGAAGCAG  R: GTGTCGCTGTTGAAGTCAGAGGAG |

**Table S5.** Antibody catalogue

| **Name** | **Catalog No.** | | **Source** | **Application/Dilution** |
| --- | --- | --- | --- | --- |
| KLF10 | 29709-1-AP | | Proteintech | WB^a)^/1:1000, IF^b)^/1:100, IHC^c)^/1:200 |
| FBXO2 | 14590-1-AP | | Proteintech | WB/1:1000, IF/1:100, IHC/1:200 IP/1:100 |
| Parkin  PINK1 | 4211  6946 | | CST  CST | WB/1:1000, IF/1:100, IHC/1:200  WB/1:1000, IHC/1:200 |
| Collagen II | 34712 | | Abcam | WB/1:1000, IHC/1:300, IF/1:100 |
| MMP-3  Aggrecan  ADAMTS-4 | | 53015  3773  185722 | Abcam  Abcam  Abcam | WB/1:1000, IHC/1:300, IF/1:100  WB/1:1000  WB/1:1000 |
| FTH | TD6278 | | Abmart | WB/1:1000, IHC/1:200 |
| GPX4 | 67763-1-Ig | | Proteintech | WB/1:1000, IHC/1: 200 |
| PTGS2 | 27308-1-AP | | Proteintech | WB/1:1000 |
| ACSL4 | 22401-1-AP | | Proteintech | WB/1:1000 |
| LCN2 | 26991-1-AP | | Proteintech | WB/1:1000, IHC/1:200 |
| LC3 A/B | 12741 | | CST | WB/1:1000, IF/1:100, IHC/1:200 |
| P62 | 88588 | | CST | WB/1:1000 |
| Beclin-1 | 3495 | | CST | WB/1:1000 |
| TOM20  4-HNE  BNIP3L/Nix  BNIP3 | 283317  PC6313  12396  44060 | | Abcam  Abmart  CST  CST | IF/1:200  IHC/1:200  WB/1:1000  WB/1:1000 |
| GAPDH  α-Tubulin  β-Actin | 60004-1-Ig  0762-1-RR  3700 | | Proteintech  Proteintech  CST | WB/1:5000  WB/1:5000  WB/1:2000 |

^a)^(Western blot);^b)^( immunofluorescence (IF));^c)^( immunohistochemistry).

**Table S6.** Primers for genotype of FBXO2

| **Primer** | **Sequence (5'-3')** |
| --- | --- |
| **Primer 1** | F1: 5’-GTAATTCCAAGGCTCAGAGCAGTA-3’ |
| **Primer 1** | R1: 5’-GATCTCTTCTGCCTATCCCAGCAT-3’ |
| **Primer 2** | F1: 5’-GTAATTCCAAGGCTCAGAGCAGTA-3’ |
| **Primer 2** | R2: 5’-ACTCCACGTCCCACTTTGAGTAAA-3’ |

**Table S7.** Sequences of adeno-associated virus 9- lipocalin 2 (AAV9-LCN2) shLCN2

| **Group** | **Target Sequence** |
| --- | --- |
| Negative control | TTCTCCGAACGTGTCACGT |
| shRNA608 | CAATGCATTGACAACTGAATG |
